# Benchmarking the performance of uncertainty quantification methods for neural network-based interatomic potentials

**DOI:** 10.1186/s13321-026-01193-7

**Published:** 2026-04-13

**Authors:** Nicholas T. Wimer, Juliane Mueller, Sebastien Hamel, Vincenzo Lordi

**Affiliations:** 1 National Renewable Energy Laboratory, Golden, CO USA; 2 Lawrence Livermore National Laboratory, Livermore, CA USA

**Keywords:** Uncertainty quantification, Neural network potential, Machine learned interatomic potential, Hyperparameter tuning, Aleatoric Uncertainty, Epistemic uncertainty

## Abstract

Machine-learned interatomic potentials (ML-IAPs) continue to gain popularity as accurate, computationally efficient replacements for traditional, physics-based interatomic potentials and expensive *ab initio* methods. Uncertainty quantification (UQ) of ML-IAPs is a growing area of research as UQ is critical in many applications of IAPs, such as developing curated datasets, active learning-based data augmentation, self-improving models, and estimating the uncertainty of molecular dynamics simulations. In this paper, we construct and benchmark a series of different neural network potentials (NNPs) with varying network architectures to determine the performance of these models with respect to both the mean and uncertainty calibration error. Each NNP method is specifically designed to predict either epistemic or aleatoric uncertainty with particular focus on the differences in behavior between the epistemic and aleatoric uncertainty estimates. We benchmark these methods using multiple datasets common in the ML-IAP literature. The results show that the aleatoric uncertainty from single-shot model architectures is a competitive alternative to ensemble-based epistemic uncertainty predictions in regions of sufficient data-density. However, in regions where the representative data is sparse, aleatoric uncertainty models tend to overpredict and epistemic methods tend to underpredict the actual model error. We conclude that the type of UQ is crucial when discussing performance of probabilistic model results as different methods have different performance characteristics depending on the regime in which they are evaluated. Therefore, the type of UQ method should be carefully evaluated against both the data characteristics and requirements for the intended application.

**Supplementary Information:**

The online version contains supplementary material available at 10.1186/s13321-026-01193-7.

## Scientific contribution

In this study, we present a systematic approach for benchmarking uncertainty quantifi cation (UQ) strategies for neural network interatomic potentials evaluated by both accuracy and uncertainty calibration metrics across multiple datasets. We present a robust hyperparameter tuning framework that allows for equitable comparison of different UQ methods and demonstrate that model performance is highly sensitive to hyperparameter settings. Finally, we conclude that when properly trained, single-shot aleatoric approaches can provide performance competitive with standard ensemble-based methods in data-rich regimes but tend to overestimate error in data-sparse regions, thereby providing guidance for UQ method selection.

**Supplementary Information:**

The online version contains supplementary material available at 10.1186/s13321-026-01193-7.

## Introduction

The development of machine-learned interatomic potentials (ML-IAPs) has enabled highly efficient and accurate simulations of high-dimensional atomistic environments which are used in a wide range of material science applications [1–3]. As machine learning (ML) methods, and in particular neural network (NN)-based methods, have rapidly developed over the last decade, so too have the capabilities of ML-IAPs. Since the key indicator of interatomic potential (IAP) performance has been the mean prediction accuracy, significant attention in the literature is devoted to examining the root mean square error (RMSE) for both energy and force predictions as well as the material properties associated with their evaluation [4–6]. While these metrics continue to be a vital measure of the success of any given IAP, additional uncertainty quantification (UQ) metrics have recently begun to garner more attention for their ability to assist in decision making processes.

When evaluating an ML-IAP on a single set of atomic structures, the model may exhibit large local errors, even if the overall RMSE of the model is low on a larger set of structures. This unforeseen error in the model’s predictions may cause issues during time-dependent atomistic simulations where such small errors in energy or force predictions can manifest as biases in quantities of interest [7]. To address these challenges, many ML-IAPs have begun to incorporate UQ techniques in an attempt to characterize the uncertainty of a model prediction and improve decision making processes [8–11]. While there are many forms of ML-IAPs, we will restrict our discussion to Behler-Parinello type neural network potentials (NNPs) [12–14].

Ensemble-based methods are among the most common approaches to UQ [15, 16]. Instead of training a single NNP, a set of NNPs can be trained and subsequently evaluated to provide ensemble statistics for each set of inputs [17, 18]. Each NN within the ensemble will have a slightly different combination of model parameters resulting in slightly different predictions. Ensemble-based approaches provide an approximation of the epistemic uncertainty, the uncertainty of the prediction due to variations in model parameters.

One of the appeals of ensemble-based methods are their conceptual simplicity, ease of implementation, and high degree of parallelism (i.e., each model can be assigned a set of computational resources and trained in parallel). There are many ways to create an ensemble. Some of the common methods are: data bootstrapping or subsampling [19], varying model weight initializations, varying model or training hyperparameters, sampling collection of model weights during training [20], Monte Carlo (MC) methods [21], and Bayesian methods [22]. Various methods of creating ensembles have all shown promising results for uncertainty calibration accuracy [23–25], and are often viewed as the benchmark against which other UQ methods should be compared.

However, despite the ease of parallelism present in ensemble-based approaches, they still require significant computational resources during both training and inference. Because of this, single-shot UQ approaches have gained popularity as they can simultaneously provide predictions along with uncertainty estimates through a single forward pass of the network. This uncertainty estimate is obtained by modeling the NNP output as a probability distribution. This uncertainty is learned during model training directly from the data and is called aleatoric uncertainty. The disadvantages of these single-shot approaches are that they require modifications to the NNP architecture and careful constructions of more complex loss functions. Recently, these models have been shown to be competitive to ensemble-based methods in their UQ performance on a variety of datasets [26, 27], but that they do not consistently outperform ensemble-based approaches [28].

In this work, we continue the investigation into the performance of single-shot UQ methods for NNPs to determine the advantages and limitations with respect to ensemble-based methods. Here, we will examine the performance of the following NNP methods in terms of energy and force RMSE and uncertainty calibration error: 1) single-model and deep-ensemble of standard artificial neural network (ANN) NNP, 2) single-model pseudo-ensemble formed via Monte Carlo dropout (MCD) layers, 3) single-shot mean-variance estimation (MVE) network, 4) single-shot mixture density network (MDN), and 5) single-model Bayesian neural network (BNN) providing an estimate of total uncertainty. Each of these methods is evaluated on multiple distinct datasets for benchmarking purposes.

The paper is organized as follows: In Sect. UQ, NNP architectures, and hyperparameter tuning, we provide a discussion on the different types of uncertainty, the various neural network architectures, and the hyperparameter tuning process; in Sect. Datasets used in numerical experiments, we describe the characteristics of the different datasets; in Sect. Results and discussion of numerical experiments we analyze the results from our numerical experiments and discuss the implications of each model’s performance; and finally, in Sect. Discussion we provide a discussion of some general observations and implications of the results; in Sect. Conclusions we summarize our work and provide an outlook on potential future directions of research.

## UQ, NNP architectures, and hyperparameter tuning

There are many methods for deriving uncertainty quantification metrics from neural networks, but they can be broadly divided into categories based on the type of uncertainty that they are attempting to approximate. These uncertainty types are: aleatoric uncertainty, arising from variability in the dataset; epistemic uncertainty, arising from variability in the model parameters; and total uncertainty, which is the sum of the aleatoric and epistemic variances. In this work, we examine ensemble-based UQ methods, providing an estimate of the epistemic uncertainty, and single-shot UQ methods, providing an estimate of the aleatoric uncertainty.

The ensemble approaches are: a deep ensemble created from top performing sets of hyperparameters and ensembles formed from multiple evaluations of a single, probabilistic model. Though different in construction, each of these ensembles provide a set of predictions from which we can calculate uncertainty metrics. Unlike the ensemble-based models, the single-shot models do not require multiple evaluations to provide an uncertainty estimate. Through a specialized output layer, both of the single-shot neural network classes we examine provide predictions as Gaussian distributions, in the case of MVE, or mixtures of Gaussian distributions, in the case of MDNs.

**Fig. 1:**
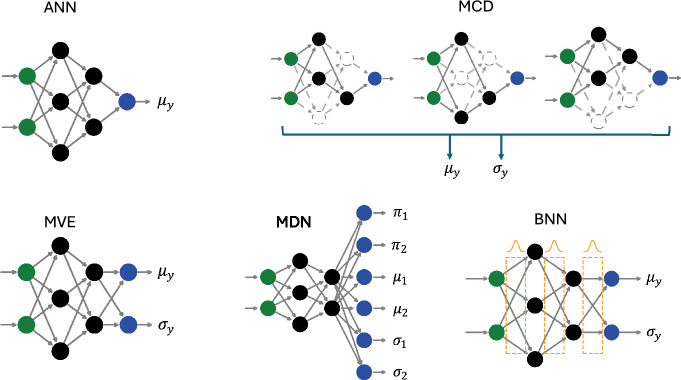
Overview of the different neural network architectures examined in this study: artificial neural network (ANN), Monte Carlo dropout (MCD), mean-variance estimation (MVE), mixture density network (MDN), and Bayesian neural network (BNN)

The model architectures, as represented in Fig. 1, are:

1. Traditional ANN with epistemic uncertainty provided by deep-ensemble statistics.
2. MCD network with epistemic uncertainty provided by ensemble statistics calculated from multiple evaluations of the same model.
3. MVE network using single Gaussian output layer to provide single-shot aleatoric uncertainty.
4. MDN network using a Gaussian mixture output layer to provide single-shot aleatoric uncertainty.
5. BNN with a single Gaussian output layer providing both aleatoric (single-shot) and epistemic uncertainty (multiple evaluations).

While common practice, using a fixed set of hyperparameters for each of these models is prone to producing suboptimal models. Since we have no reason to expect each of these model classes to respond similarly to identical hyperparameters, we first perform an extensive hyperparameter tuning process for each of the models. In this section, we provide a discussion on the different types of uncertainty, describe the models trained in this study, and discuss the hyperparameter tuning process.

### Uncertainty quantification (UQ)

In general, there are two forms of uncertainty, aleatoric and epistemic [29]. The aleatoric uncertainty is an estimate of the uncertainty contained in the data and the epistemic uncertainty is an estimate of the uncertainty due to model parameters. The epistemic uncertainty can be estimated for every model architecture by forming a deep-ensemble from a set of NNPs with varying model parameters. Estimating the aleatoric uncertainty, on the other hand, requires specific architecture features, such as the addition of a custom output layer either in the form of a simple MVE or the more general MDN output. Some models, such as MCD networks and BNNs contain NN layers specifically designed to capture variability in model parameters and provide epistemic uncertainty estimates via pseudo-ensemble: multiple forward passes of a single network.

In the case of NNP capable of providing estimates of both aleatoric and epistemic uncertainty, the total uncertainty is computed as the sum of the two variance estimates according to the law of additive variance. In the case of the BNN examined here, the epistemic uncertainty comes from the sampling of the posterior distribution over the weights and the aleatoric uncertainty comes from direct approximation through a MVE output layer. The total uncertainty is computed as:

1 $$\begin{aligned} ~ \sigma _{total} = \sqrt{\sigma ^2_{epistemic} + \sigma ^2_{aleatoric}} \,. \end{aligned}$$

In an ideal scenario, the aleatoric and epistemic components of a model’s uncertainty prediction would be independent of one another and the total uncertainty (Eq. 1) would contain no redundancy. In practical applications in NN approaches, this is rarely observed to be the case, with aleatoric and epistemic uncertainty becoming coupled and even one uncertainty type co-opting the other during training [30]. Therefore, for highly non-linear NN applications, special care must be applied to disentangle uncertainty estimates if separate, pure aleatoric and epistemic are needed [31]. Here, we present the total uncertainty with the understanding that it might contain redundancy and therefore be an overestimate of total uncertainty.

In the context of uncertainty-aware NNPs, there are two uncertainty metrics that we consider in this study: the uncertainty of the total energy and the uncertainty of the atomic forces within a single atomic structure. An NNP makes predictions of the atomic energies which are summed for each atom in the atomic structure to give the total energy. Gradients of resulting total energy are then used to compute the forces per atom in an energy conserved manner. The NNPs that attempt to estimate aleatoric uncertainty (e.g., MVE, MDN, and BNN) will also directly provide predictions of the atomic variances in both total energy and atomic forces independently. While it is possible for the NNP to compute a single energy variance and directly compute variances in the force components through careful propagation of uncertainties through the gradients, this leads to a significant increase in the computational cost of the NNP. To avoid this spike in computational cost for aleatoric uncertainty, our framework provides quantification of the aleatoric uncertainty for both the total energy and the atomic force independently. This framework results in direct force uncertainty estimates for each force component for each atom and a separate, uncoupled total energy uncertainty estimate for the atomic structure. The force uncertainty estimates are direct outputs from the NNP and the computation of these values is detailed in the loss functions for the different methods below. When computing statistics for force uncertainties, one value is reported after aggregating all components together, similar to what is commonly done for RMSE. For example, in a standard MVE implementation, there are four distinct variances trained for each NNP (one for total energy and three for the force components). More information regarding the specifics of uncertainty implementation for each model type is contained in Sects. Ensemble methods – Bayesian neural networks (BNNs).

The aleatoric variance of the total energy of a single atomic structure is computed as the sum of the variances for each atomic energy prediction within that structure:

2 $$\begin{aligned} \sigma ^2_{E\text {, aleatoric}} = \sum _i{\sigma ^2_{e_i}} \,, \end{aligned}$$

where $\sigma ^2_E$ is the total energy variance in the atomic structure and $\sigma ^2_{e_i}$ is the variance in the energy prediction for each atom within the structure.

The epistemic variance, on the other hand, is computed directly as the variance of the total energy prediction accumulated over each atomic structure:

3 $$\begin{aligned} \sigma ^2_{E\text {, epistemic}} = \frac{1}{M} \sum _{m=1}^{M} (E^{(m)} - \bar{E})^2 \,, \end{aligned}$$

where $E^{(m)}$ is the total energy prediction for a given model, *M* is the number of models in the ensemble, and $\bar{E}$ is the average total energy prediction across the ensemble of models.

### Ensemble methods

Ensemble methods utilize a set of model predictions to derive a statistical distribution of output values given a single input. Although the formation of the ensemble differs by implementation, the final prediction and its uncertainty are computed as the arithmetic mean and standard deviation of the ensemble predictions. The size of the ensemble required to provide a reliable estimate of the uncertainty is dependent upon the underlying “true” uncertainty (although some studies suggest an ensemble size on the order of tens to hundreds of members is sufficient [32–34]). If each of the models within the ensemble are trained using either different sections of the data, different random weight initializations, or even different model architectures, each of the models will exhibit various performance tradeoffs for different sections of the input data, even if achieving the same RMSE on the full dataset. This spread in the disagreement between the different models gives an approximation of the epistemic uncertainty.

While ensemble methods can explicitly train *M* different models, pseudo-ensembles can also be created using a single model that provides non-deterministic predictions (i.e., each evaluation of the model yields a different output). If the model provides independent samples from a representative probability density function (PDF) every time that it is queried, a pseudo-ensemble of size *M* can be obtained by sampling from the probabilistic model *M* times. In this study, we examine two such model architectures capable of obtaining pseudo-ensembles via probabilistic predictions. These methods are integrated directly into the architecture of the NNP by means of MCD and Bayesian flipout layers [35]. These two layer types enable probabilistic predictions with each forward pass through the network and therefore form an ensemble after *M* independent evaluations. This approach makes obtaining an ensemble evaluation of the dataset more straightforward and reduces the complexity of individually training *M* separate models and storing/loading/evaluating *M* sets of NN weights. Both of these methods are discussed in more detail below.

### Monte Carlo dropout (MCD)

MCD is a technique for creating a pseudo-ensemble by training a single NN model with at least one dropout layer. In typical applications of dropout layers, the dropout layer is only active during model training, which helps to stabilize the training process and prevent over-fitting by randomly bypassing a percentage of the model weights during each iteration. To then generate a probabilistic model that was trained using dropout, the NN model simply needs to enable those dropout layers during inference. Since dropout layers are routinely included in a variety of model architectures for regularization, MCD is one of the simplest ways of creating a pseudo-ensemble from a single NN architecture. The pseudo-ensemble is generated by evaluating the model *M* times; due to the dropout layer(s), each pass through the network forms a deterministic sub-net of width: $w_s = w_f * (1 - d)$, where $w_f$ is the full width of the layer and *d* is the dropout rate. Each sub-net that is evaluated during the forward pass is itself deterministic (i.e., will always provide the same prediction given the same input and subset of active neurons). The selection of the active neurons is, however, probabilistic and the width of the resulting sub-net directly related to the dropout rate. Because of this, the estimation of epistemic uncertainty gained from MCD techniques is highly dependent upon the selected dropout rate and is therefore included in the set of hyperparameters during model tuning.

The loss function used for both the standard ANN and MCD models is the standard mean squared error (MSE) loss in terms of both total energy and atomic forces per structure:

4 $$\begin{aligned} ~ \mathcal {L}_{\text {MSE}} = (E - \hat{E})^2 + \sum _i (F_i - \hat{F_i})^2 \,, \end{aligned}$$

where *E* and $F_i$ represent the true total energy and force components, respectively and $\hat{E}$ and $\hat{F_i}$ represent the predictions. Note that we do not perform a weighted average and treat contributions from both quantities equally.

### Mean-variance estimation (MVE)

One of the more straightforward ways to directly incorporate the aleatoric uncertainty of a model prediction is through direct modeling of the mean and variance of the input data. Although mean-variance estimation can be applied across a broad range of approaches, here we refer to MVE as a specific neural network model architecture. Since the learned variance, as with the mean prediction, is a function of the input parameters, MVE is capable of representing heteroscedastic uncertainty. To implement MVE within a NNP, the underlying architecture is modified to separately provide output nodes that are mapped to the mean and variance via a loss function that typically assumes a normal distribution. This allows the NNP to learn the appropriate mean and variance in both energy and forces as a function of atomic structure.

Instead of providing a single output for each dependent variable, the MVE doubles the number of outputs to 2*N*, where *N* is the number of output parameters. Each set of outputs represents the mean and variance of the dependent variable with respect to the model inputs. For the MVE to learn the mean and variance of the outputs, a form of the underlying PDF must be presumed and formulated into a loss function.

A common choice for the underlying PDF is a Gaussian distribution with uncertainty symmetrically distributed about the mean. The Gaussian PDF is given by:

5 $$\begin{aligned} p(y | x) = \frac{1}{\sqrt{2 \pi \sigma ^2(x)}} \exp \left( -\frac{(y - \mu (x))^2}{2 \sigma ^2(x)} \right) \,, \end{aligned}$$

where *p*(*y*|*x*) is the probability of the output *y* given the input *x*, $\sigma (x)$ is the standard deviation of the output with respect to the input, and $\mu (x)$ is the mean prediction with respect to the input.

By presuming the Gaussian distribution of the uncertainty, the negative log likelihood (NLL) loss function for a MVE output layer is derived by taking the negative logarithm of the Gaussian PDF (Eq. 5). Here, we use a modified version of the pure NLL loss function that includes a MSE regularization term:

6 $$\begin{aligned} \mathcal {L}_\text {MVE}&= \frac{(E - \mu _E)^2}{2 \sigma _E^2} + \frac{1}{2} \log (2\pi \sigma _E^2) + \lambda (E - \mu _E)^2 \nonumber \\&\quad + \sum _i \left[ \frac{(F_i - \mu _{F_i})^2}{2 \sigma _{F_i}^2} + \frac{1}{2} \log (2\pi \sigma _{F_i}^2) \right] \,, \end{aligned}$$

where $\lambda$ is an optional weighting term for the regularization. Since the terms associated with the mean prediction accuracy contain variances in the denominator, one mode of minimizing the above equation is to increase the variance in the prediction, oftentimes resulting in poor mean performance and uncalibrated uncertainty estimates. To avoid this mode of failure, an additional MSE term is added to the loss function which acts to regularize the NLL loss. While this regularization term can be applied to both energy and forces, we found that regularizing just the energy term, with $\lambda = 1$, was sufficient to prevent the runaway variance effect. Since the variance is learned directly from the distribution of the data itself, the MVE network provides an approximation of the aleatoric uncertainty.

### Mixture density networks (MDNs)

MDNs are a natural extension of the standard MVE; while a simple MVE models the output as a single Gaussian PDF, MDN models the output as a weighted average of *m* independent Gaussian PDFs [36]. Explicitly, the underlying PDF of a Gaussian MDN is of the form:

7 $$\begin{aligned} p(y | x) = \sum _{i=1}^m \pi _i(x) \cdot \frac{1}{\sqrt{2 \pi \sigma _i^2(x)}} \exp \left( -\frac{(y - \mu _i(x))^2}{2 \sigma _i^2(x)} \right) , \end{aligned}$$

where $\pi _i(x)$ is the mixing coefficient for the *i*-th Gaussian, such that $\sum _{i=1}^m \pi _i(x) = 1$, $\mu _i(x)$ is the mean of the *i*-th Gaussian, and $\sigma _i^2(x)$ is the variance of the *i*-th Gaussian.

The output layer of an MDN requires expanding the number of neurons from *N* for a simple ANN to $3N \times m$ to account for each distribution’s mixing coefficient, mean, and variance (or at a minimum $m(3N-1)$ if we enforce $\pi _{m}(x) = 1 - \sum _{i=1}^{m-1} \pi _i(x)$). Additionally, a softmax activation function is applied across the output of the neurons representing the mixing coefficients to ensure that they sum to one.

The loss function is similarly derived as a modified NLL loss function by taking the negative log of the mixture density function. The resulting MDN loss in terms of NNPs is:

8 $$\begin{aligned} \mathcal {L}_\text {MDN}&= - \log \Bigg ( \sum _{j=1}^m \pi _j \cdot \exp \Bigg ( - \Bigg [ \frac{(E - \mu _{E_j})^2}{2 \sigma _{E_j}^2} + \frac{1}{2} \log (2 \pi \sigma _{E_j}^2) \nonumber \\&\quad + \sum _{i=1}^3 \Bigg ( \frac{(F_i - \mu _{F_i,j})^2}{2 \sigma _{F_j}^2} + \frac{1}{2} \log (2 \pi \sigma _{F_j}^2) \Bigg ) \Bigg ] \Bigg ) \Bigg ) \nonumber \\&\quad + \lambda (E - \mu _E)^2 \,. \end{aligned}$$

Similar to the MVE, the mean prediction performance can sometimes be degraded by an ever increasing uncertainty prediction, therefore a MSE loss is added to the resulting MDN loss as a regularization term to improve performance. Since the MDN is able to learn a mixture of Gaussian distributions, the resulting NN should be better suited to approximate multi-modal trends in the data and/or its variance [36]. As with the MVE, the MDN provides a direct estimate of the aleatoric uncertainty.

### Bayesian neural networks (BNNs)

BNNs extend the traditional ANNs by learning a full PDF for each of the network’s weights and biases instead of a deterministic value [37]. Every node in the network contains PDFs that represent the prior and posterior distributions of the node’s weights and biases. By learning a PDF for the weights and biases, BNNs directly quantify the epistemic uncertainty as they capture the distribution of the learned weights. The BNN posterior distributions over the weights and biases are all updated using Bayes’ theorem:

9 $$\begin{aligned} p(W | D) = \frac{p(D | W) p(W)}{p(D)} , \end{aligned}$$

where *p*(*W*|*D*) is the posterior distribution of the weights *W* given the dataset *D*, *p*(*D*|*W*) is the likelihood of data given a set of weights, *p*(*W*) is the prior distribution over the weights, and *p*(*D*) is the marginal likelihood.

BNNs can require a significant amount of computational overhead due to the probabilistic framework of the network parameters. Since directly computing the posterior distribution is often computationally intractable for modern deep networks, BNNs often use approximate techniques such as variational inference (VI) or Markov chain Monte Carlo methods for approximate Bayesian methods.

In this work, we implement a BNN using DenseFlipout [35] layers which are a specific implementation of VI that can efficiently estimate the uncertainty in the weights while reducing noisy gradient updates which can impede learning. Specifically, the DenseFlipout layer introduces a pseudo-independent noise to each weight perturbation; this mitigates variance inflation and overall improves training stability. The weight update is given as:

10 $$\begin{aligned} W = \mu _W + \Delta W \cdot r_1 + \Delta W^T \cdot r_2 , \end{aligned}$$

where $\Delta W$ is the sampled perturbation, $r_1$ and $r_2$ are randomly sampled sign matrices that provide the independent noise realizations for each input sample. With this formulation, each forward pass through the network represents a different realization of the underlying posterior distribution which forms a pseudo-ensemble from a single model given *N* independent evaluations, similar in theory to the MCD method. The ensemble mean again serves as the final prediction and the variance across the ensemble represents the epistemic uncertainty. However, unlike MCD, which relies on random neuron deactivation per layer, DenseFlipout explicitly models the epistemic uncertainty during the training process.

BNNs can be combined with a MVE or MDN output layer which allows the BNN formulation to provide a joint quantification of the aleatoric and epistemic uncertainty. The BNN we use contains a MVE output layer, extending the network to learn the aleatoric uncertainty while retaining the epistemic uncertainty estimate native to the BNN formulation.

### Hyperparameter tuning

As with any ML method, the choice of hyperparameters can play a significant role in the quality of the results. To aid in the search of the sets of hyperparamters which produce the very best results, we use an automated hyperparameter tuning approach implemented in Ray Tune [38], a Python library developed for scalable hyperparameter tuning.

Within Ray Tune, there are many different approaches that can be used for searching the hyperparameter space. For our study we use Optuna [39] with a Tree-structured Parzen Estimator (TPE) sampler [40] and an Asynchronous Successive Halving Algorithm (ASHA) scheduler [41]. Table 1 summarizes the settings that were used for the hyperparameter tuning for each of the two datasets examined in this study. These settings themselves can have an impact on the overall tuning process. We hand-selected these values after coarse, non-exhaustive experimentation. The differences between the Ray Tune settings between the two datasets is due to differences in the total number of atoms in each dataset, necessitating fewer number of total models and fewer map epochs with the GAP-20 dataset.

**Table 1:** Ray Tune settings for the MLEARN and GAP-20 datasets

Setting	MLEARN Value	GAP-20 Value
Sampler	TPE	TPE
Scheduler	ASHA	ASHA
Number of samples	500	250
Number of concurrent trials	100	10
Max Epochs	10,000	1,000
Grace Period	100	100
Reduction Factor	4	4
Number of brackets	2	2

Since the different neural network architectures have different sets of relevant hyperparameters, the number of tunable parameters will vary with method. Table 2 summarizes the range of settings that were used for each of the datasets to determine the best sets of hyperparameters for the final models. The baseline settings represent the set of hyperparameters that are consistent across each model type. All other unique hyperparameters and their associated ranges are provided. All of the models were trained with a starting learning rate of 1e-3 and using a dynamic learning rate scheduler that reduces the learning rate by a factor of two once the validation loss plateaus. The hyperparameter optimization routine minimizes the validation loss as the objective function. While this is standard practice, explicit UQ performance criteria could also be minimized, which might yield more desirable results depending on the application and intended use-case.

**Table 2:** Collection of hyperparameter settings used for searching the hyperparameter space for all NNP models. Each of the models share the settings listed under ‘Baseline Settings’ as well as the additional settings under their respective model types

Hyperparameter	Value
*Baseline Settings*	
Activation Function	{TanH, GeLU, Leaky ReLU, Swish}
L2 regularization	[1e-6, 1e-3]
Neurons per block	{0, 2, 4, 8, 16, 32, 64, 128, 256}
Max number of blocks	2
Layers per block	2
*MCD Settings*	
Dropout	[0.1, 0.5]
*MVE and BNN Settings*	
Mixtures	1
*MDN Settings*	
Mixtures	[2 : 10]

**Fig. 2:**
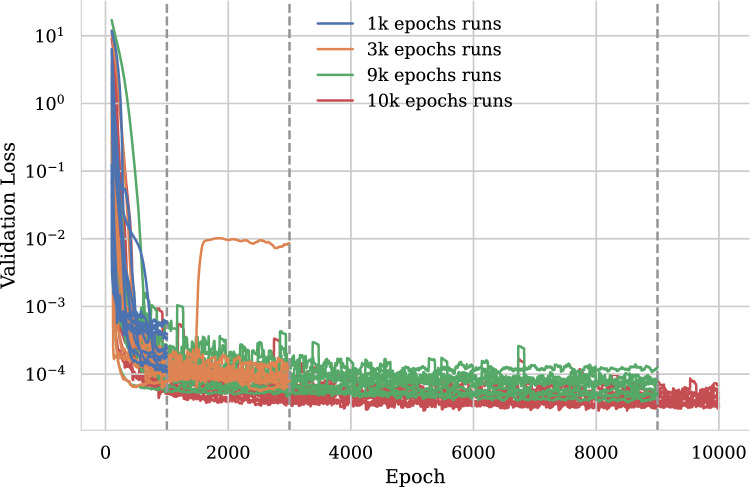
Validation loss as a function of training epoch from a subset of NNPs trained using the hyperparameter tuning framework. Sample runs are shown from the subset of runs that were terminated at 1k, 3k, 9k, and 10k epochs. Only the best performing runs are considered for the deep ensemble

The hyperparameter tuning settings in Table 1 result in a parameter search that performs early termination of models at the following intervals: 1k, 3k, and 9k epochs. At these points during the training process, under-performing models are dropped from the training sweep and computational resources are devoted to training only the best performing models up to the maximum 10k epochs. Figure 2 shows the validation loss curves for a subset of the models trained for each of these epoch intervals. At the end of training, a deep ensemble can be created by taking the top *N* models with the lowest validation loss. By forming the ensemble during the hyperparameter tuning process itself, each member of the ensemble is highly performant and the ensemble is created with no additional computational expense compared to the tuning process itself.

### Implementation details

All models in this work are Behler-Parrinello type NNPs using atom-centered symmetry functions (ACSF) descriptors to represent local atomic environments. All of the NNPs are implemented using the KIM-based Learning-Integrated Fitting Framework (KLIFF) framework [42], which was built to provide a module environment for constructing and fitting NNPs. KLIFF handles the configuration of the datasets, the generation of the ACSF descriptors from atomic position files, formulation of the loss functions, and performing the model training. All of the ACSF descriptors use a set of 51 symmetry functions with a cosine cutoff and symmetry normalization. The ACSF descriptors use different cut distances for the different species studied: {Cu, 4.1}; {Ge, 5.6}; {Li, 5.2}; {Mo, 5.2}; {Ni, 3.9}; {Si, 5.5}; and {C, 4.5}. The underlying NN architectures and handling of the UQ metrics are built using the PyTorch Machine Learning Toolbox (PT-MELT) [43], a Python package designed to streamline the development of scientific ML models with native support for probabilistic NNs including MVEs, MDNs, and BNNs. The combined framework allows for direct integration of all the NNPs described in this section as well as the means for monitoring the training, hyperparameter tuning, and evaluating the model’s prediction and UQ metrics.

## Datasets used in numerical experiments

**Fig. 3:**
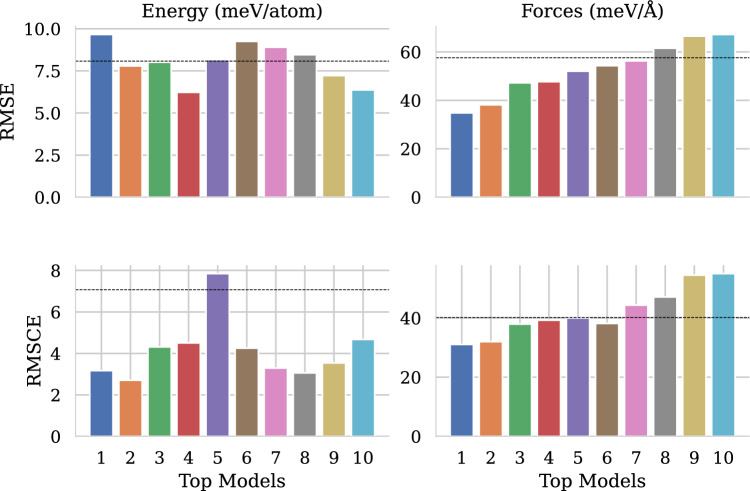
Distribution of training, validation, and test frames as a function of element or structure types for the MLEARN and GAP-20 datasets, respectively. Each element of the MLEARN dataset contains similar numbers of frames, whereas the GAP-20 dataset shows a large disparity between structure types. Note the linear scale on the MLEARN plot and the log scale on the GAP-20 plot

We use several distinct datasets to evaluate the performance of the NNPs. The datasets are chosen to provide an evaluation on a simpler case that has well established performance benchmarks and a larger, more complex case to stress test the various methods on a richer set of structures. Figure 3 shows the distribution of the training, validation, and test splits for each element or structure contained within the datasets. The MLEARN datasets provide roughly an equal number of frames per element, whereas the GAP-20 dataset has a large range of frames for each of the human labeled component structures.

### MLEARN dataset

The MLEARN dataset is described in Zuo et al. and provides data and benchmarks for Cu, Ge, Li, Mo, Ni, and Si for a variety of IAPs including NNPs and traditional methods [44]. This dataset is published online, alongside the paper, with both training and test data splits for the evaluation of ML-IAPs. This dataset has well-established benchmarks for performance provided in the original work as well as subsequent studies by a variety of authors [45–47]. Here we will evaluate our own methods on these popular benchmarks and provide the performance of the models with respect to uncertainty calibration as additional benchmarks.

All elements in the MLEARN dataset contain at least 150 atomic structures for six different elements. Each of the elements are divided using an 80-10-10 percentage split between the training, validation, and test set, respectively. The models are only trained on the training split, the validation split is unseen during model training, but is used as the performance metric during hyperparameter tuning. The test set is completely unseen during both training and tuning and is used as the ultimate benchmark for model performance. All results presented are in terms of performance on this test data split.

### Carbon GAP-20 dataset

The second dataset is a resampled version of the GAP-20 carbon dataset [48]. This dataset is substantially larger than the previous, consisting of a total of 6066 frames representing 17 “different” carbon structures relaxed using density functional theory (DFT) simulations. The internal divisions within the GAP-20 dataset were determined via human labeling and may not perfectly align with clean divisions in atomic structure or resulting properties. The overlap between these divisions is discussed in more detail in Schwalbe-Koda et al. [49]. Figure 3 shows the breakdown of the different structures contained in the GAP-20 dataset along with their representation within the train, validation, and test datasets used within this study. The same 80-10-10 split was used independently for each of the structures, prioritizing at least one representative frame from each structure be contained within the test split of the data. For certain structures (e.g., dimer, graphene, graphite layer sep, and liquid), there is an insufficient number of frames to a full 80-10-10 split. For these structures, frames were excluded from the validation set in lieu of more frames included in both the train and test splits.

The majority of the structures contained within the GAP-20 dataset are described as ‘amorphous bulk’. Some of the least represented structures are: ‘amorphous surfaces’, ‘dimer’, ‘graphene’, and ‘liquid’. The over-representation of one structure type compared to the rest (particularly within) the training portion of the dataset, has the potential to bias the model dynamics to represent amorphous bulk structures. If this is the case, we would expect to see better performance in these over-representated structures, worse performance in the under-represented structures, and a corresponding increase in the uncertainty associated with under-represented structures. We discuss the performance on each of the structure types within the GAP-20 dataset in Sect. GAP-20 dataset results.

## Results and discussion of numerical experiments

The results for each of the different NNPs trained with the various underlying neural network architectures are presented below for both the MLEARN and GAP-20 datasets (described in Sect. Datasets used in numerical experiments). There are five different model architectures (ANN, MCD, MVE, MDN, and BNN), six elemental datasets included in the MLEARN paper, and 17 different structures contained in the recomputed GAP-20 dataset. For each element in the datasets (i.e., Cu, Ge, Li, Mo, Ni, Si, and C) we trained ensembles of NNPs spanning a wide range of hyperparameter settings. In total, we trained 35 different NNP ensembles (5 models across 7 elemental datasets), each formed by the Ray Tune hyperparameter tuning algorithm that spans either 500 hyperparameter sets for the MLEARN elements, or 250 hyperparameter sets for carbon GAP-20, respectively. In total, we screened 16,250 NNPs (5 models across 6 elements at 500 hyperparameter sets plus 5 models for carbon at 250 hyperparameter sets). The results for the best performing sets of NNPs in each of the relevant categories are presented below.

For each of the datasets, the mean prediction performance is determined by the best single-performing model for each NNP type. For the ANN type model that does not provide any native, single-model UQ, an ensemble of the best 10 performing models is used to compute epistemic uncertainty and associated performance metrics.

In the following we examine the model types: 1) ANN with mean performance determined by the single best performing model and epistemic uncertainty computed using an ensemble of the top 10 best performing models, 2) MCD with mean and epistemic uncertainty determined by the single best performing model evaluated 10 times to form a pseudo-ensemble, 3) MVE with mean and aleatoric uncertainty determined by a single-shot evaluation of the best performing model, 4) MDN with mean and aleatoric uncertainty determined by a single-shot evaluation of the best performing model, and 5) BNN with mean and total uncertainty determined by 10 evaluations of the same single model. In the case of the BNN, the aleatoric uncertainty component is formed using the average of the aleatoric uncertainty predictions from the pseudo-ensemble.

### Ensemble performance

**Fig. 4:**
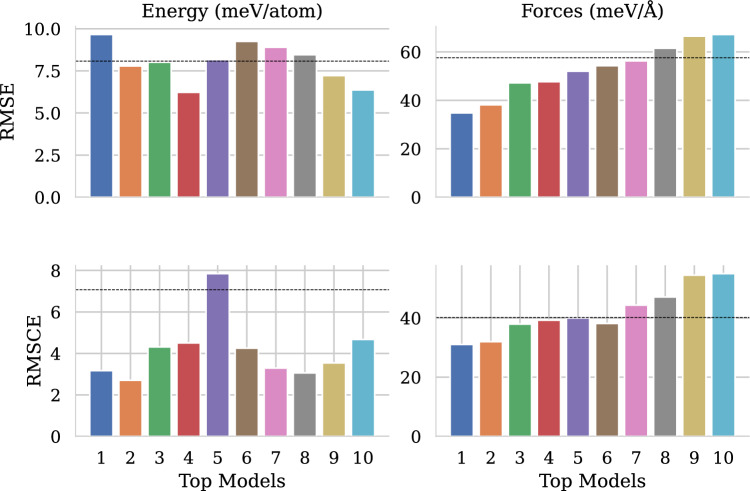
Energy and Forces performance of the top 10 individual MVE models within the deep ensemble trained on the Cu dataset. Dashed lines indicate the performance of the ensemble. The models are sorted in ascending order solely based on RMSE of atomic forces, with lower values indicating higher mean calibration accuracy

Since we optimized each model using the hyperparameter approach discussed in Sect. Hyperparameter tuning, there are hundreds of candidate models per element that could be evaluated for each NNP type. As a representative example of ensemble performance for a subset of these NNPs, Fig. 4 shows the performance of the top 10 models trained for the copper portion of the MLEARN dataset using the MVE model type. The MVE model type was chosen as a representative example because it provides a native aleatoric uncertainty estimate and therefore the uncertainty calibration performance for each of the individual models can be compared to that of the entire ensemble (the same could also be done for the MDN or BNN methods, but MVE is presented for simplicity). Both the RMSE and root mean square calibration error (RMSCE) are shown for both total energy predictions and atomic force predictions. The RMSCE is defined as:

11 $$\begin{aligned} \text {RMSCE} = \sqrt{ \frac{1}{N} \sum _{i=1}^{N} \left( \sigma _i - \left| \hat{y}_i - y_i \right| \right) ^2 } \end{aligned}$$

where $\sigma _i$ is the uncertainty prediction for a given frame, $y_i$ is the true value, $\hat{y_i}$ is the predicted value (either forces or total energy), and *N* is the number of atomic structures (in the case of total energy), or atoms (in the case of atomic forces), depending on the quantity of interest. This calibration error represents the degree to which the predicted uncertainty (in this case aleatoric uncertainty) agrees to the actual error of the mean prediction with respect to the truth value.

The dashed line in Fig. 4 represents the RMSE of the predictions derived from the ensemble. Each ensemble prediction is calculated as the mean of the predictions from each of the members of the ensemble set. Many studies have found that ensemble predictions can often outperform predictions from single models on a variety of machine learning tasks [15]. In our investigation, we found that the single best model from the hyperparameter tuning process was better, on average, than that of the top 10 ensemble. Figure 4 shows that while the mean of the ensemble is able to achieve relatively accurate predictions, the best performing single-model in each category is always able to outperform the ensemble mean. We do note that model #1 outperforms the ensemble mean on all metrics except for the total energy RMSE. For this reason, in the following sections, the ensemble mean values are only used when necessary to provide an uncertainty estimate, which is only true for the ANN model type which does not provide native UQ. For all other model types, the performance of the single-best performing model is reported.

### MLEARN dataset results

**Fig. 5:**
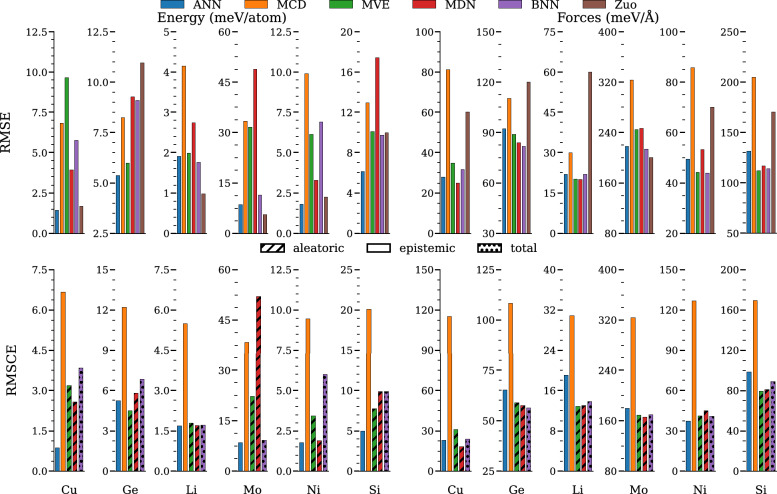
RMSE and RMSCE test results from the deep ensembles trained on the MLEARN dataset. The top panel shows the RMSE results for both energy (meV/atom) and forces (meV/Å) for each of the model types and Zuo et al. [44], for reference. The bottom panel shows the RMSCE results for each of the models presented with bars shaded based on the representative uncertainty type (e.g., epistemic, aleatoric, or total uncertainty)

The MLEARN dataset is well documented in the literature with Zuo et al. providing RMSE performance benchmarks for a variety of ML-IAPs [44]. This level of benchmarking makes it particularly attractive to use as a test case for new methods. Figure 5 shows the performance of each of the five model types for all six elements in the MLEARN dataset with respect to both RMSE and RMSCE for both total energy and atomic forces on the test dataset. The RMSE results are all shown for the single best performing model within each model type. The results for the RMSE from the NNP reported in Zuo et al. are shown as a reference benchmark. The RMSCE results are likewise shown for the single best model with the exception of the ANN results which require the evaluation of the deep ensemble to compute uncertainty. The bars within the figure are colored by the model type and shaded based on the uncertainty type used in the calibration: epistemic for ANN and MCD, aleatoric for MVE and MDN, and total uncertainty for the BNN.

Overall, we see that the mean performance of each of the models is comparable to established baselines. We note that the performance of each of our models tends to be more accurate with respect to atomic forces than with respect to the total energy. For example, the best performing NNPs on the lithium portion of the dataset can achieve a force RMSE of approximately 20 meV/Å which is significantly lower than the MLEARN published result of 60 meV/Å, but the same models struggle to maintain energy RMSEs below 2 meV/atom, whereas the MLEARN reference model achieves 1 meV/atom. The tradeoff between energy and force accuracy can be influenced by modifying the loss function (Eq. 4) to perform a weighted average of the component terms. The choice of weights can be seen as an additional hyperparameter for model selection, but we do not include this in our optimization. For simplicity, we equally weight the contribution from energy and forces. Results would likely change if a weighted loss function were used, though we do not examine that here.

In terms of total energy RMSE performance, the ANN model type consistently performs the best and the second best model types are either the MVE, MDN, or BNN. The MCD model is routinely one of the worst performing for total energy RMSE. In terms of atomic force RMSE, we do not observe a single model type that consistently outperforms the others, although we note that the ANN is never the best performing model with respect to atomic forces on the test set. Instead, the MVE model is best for silicon, the MDN is best for copper, the BNN is best for germanium and molybdenum, and both MVE and MDN are equally competitive for lithium, and MVE and BNN are equally competitive on the nickel dataset.

Also of note is that the ANN models perform better than the benchmark Zuo et al. models (which are also ANN type) for all energy RMSEs except for lithium and molybdenum and all force RMSEs except molybdenum. While it is possible that these performance improvements could be attributed to the robust hyperparameter tuning for the present models, it could also be a number of other contributing factors such as to training methodology or NNP implementation.

When considering the calibration errors, there are no performance benchmarks for reference. In terms of total energy RMSCE performance, we observe that the epistemic uncertainty estimate provided by the deep ANN ensemble is consistently one of the lowest calibration errors. The main exception to this is on the germanium data where the MVE’s aleatoric uncertainty is lowest. On the lithium, molybdenum, and nickel data, Fig. 5 shows that the aleatoric and total uncertainty predictions can be equally as calibrated as the deep ensemble.

For force RMSCE, the data show that the uncertainty estimates from the deep ensemble are still quite low, however, there is routinely an estimate from a single-shot model that is equal to or lower than that of the ensemble. The MDN has the best performance for copper and molybdenum, the MVE has the best performance for lithium and silicon, the BNN has the best performance on germanium, and the ANN is best for the nickel data. On average, the MCD performance is worse than each of the other methods.

**Fig. 6:**
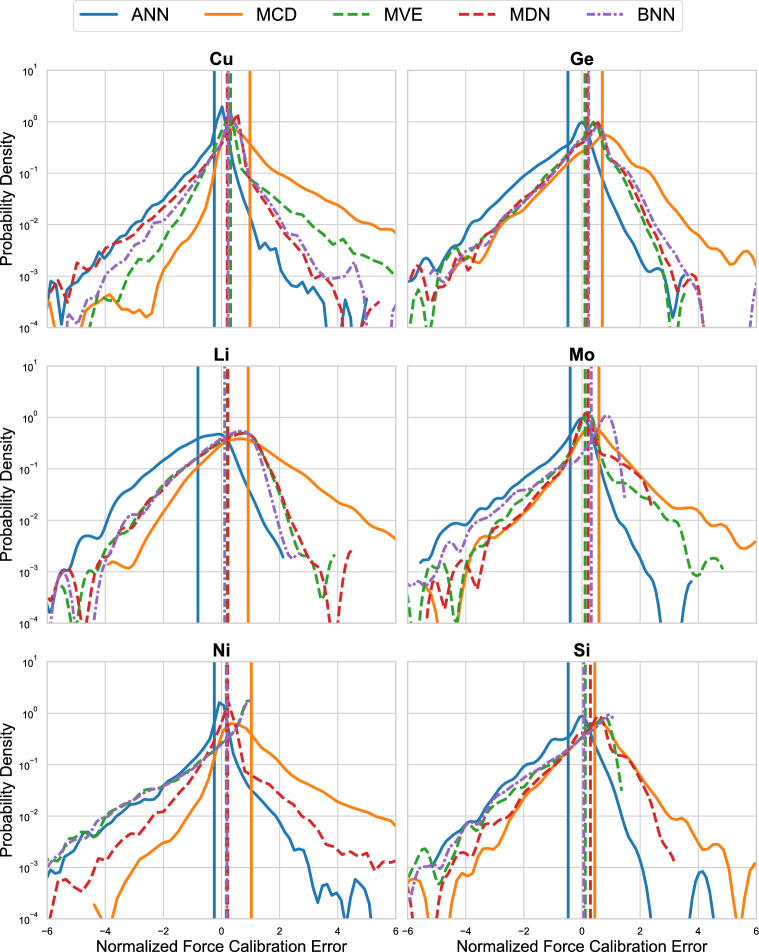
The PDFs of the performance on different elements of the MLEARN dataset for the different NNP methods. The PDFs show the normalized force calibration error with positive values corresponding to overprediction and negative values corresponding to underprediction of the true force error. The solid, dashed, and dotted lines represent epistemic, aleatoric, and total uncertainty metrics, respectively. The vertical lines denote the means for each method

While the RMSCE values are useful for determining the performance of each of the different methods on average, they do not provide information on outliers or the direction of any miscalibration in uncertainty estimates. Figure 6 shows the normalized force calibration errors as PDFs for each of the different UQ model methods for each element within the MLEARN test dataset (the same test data as in Fig. 5). The PDFs provide more structured information regarding how each of the models perform with respect to the reliability of the provided uncertainty estimates. Better performing models have a narrow distribution of force calibration error symmetrically centered around zero. Deviations from symmetry represent the model’s tendency to over- or under-predict the true error in the force calibration, indicating a bias that should be characterized to better inform UQ-based decision making. In Fig. 6, we indicate the mean of each of the PDFs using vertical lines to better indicate these trends.

For all of the elements in the MLEARN dataset, we observe that the epistemic uncertainty estimate provided by the ANN deep ensemble tends to favor underpredicting the true error associated with the mean prediction. This observation is in contrast with the epistemic uncertainty provided by the MCD single-model pseudo-ensemble which consistently overpredicts the true force error. The aleatoric uncertainty estimates from the MVE and MDN networks display more symmetric behaviors on average with mean values close to zero, albeit a slight bias for error overprediction. Finally, the total uncertainty estimate from the BNN does not outperform the aleatoric uncertainty estimates, nor does it appear to be a significant underperformer on any of the elements within the MLEARN dataset. For all elements contained within the MLEARN datasets, we observe that the ANN models always display the lowest mean normalized force calibration error, followed by a tight grouping of the aleatoric enabled models (e.g., MVE, MDN, and BNN), with the MCD model consistently displaying the largest overprediction of uncertainty.

The main observations from Figs. 5 and 6 are: the ANN-based deep ensembles and the associated epistemic uncertainty provide robust performance in terms of energy/force RMSE and energy/force RMSCE, but tend to provide a systematic underestimation of the true error; MCD-based single-model evaluations are highly variable and tend to provide poor performance compared to the other models; the aleatoric and total uncertainty estimates provided by single-model approaches (e.g., MVE, MDN, and BNN) provide consistently high performance in particular on force predictions, often achieving lower errors than their deep-ensemble counterparts; and finally, while general trends are identified, these results remain dependent on the specific dataset used as there is no clear, overall best single model type for every category examined. This type of “no free lunch”, commonly observed in multi-objective optimization problems, motivates the need to carefully characterize different types of models on relevant performance metrics prior to deploying on a new dataset.

### GAP-20 dataset results

The results presented in Sect. MLEARN dataset results are derived from training models on datasets with relatively uniform atomic structure characteristics associated with each of the individual element types. The GAP-20 dataset, however, consists of a variety of human-labeled carbon structures with large discrepancies in data representation among its component structures. Figure 3 shows this large difference in data structures between the training, validation, and test sets. In this section, we analyze the same properties of the different NNPs, with specific attention paid to the differences in performance behavior that can arise when there is unequal representations of atomic structures within a single dataset.

**Fig. 7:**
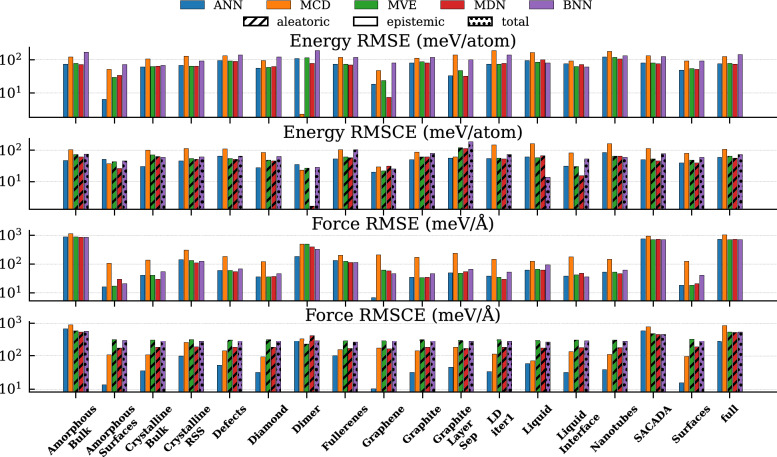
Results for the evaluation of the different NNP model types on the testing dataset of GAP-20. The results show energy and forces RMSE and RMSCE with patterns to indicate the different UQ types (i.e., epistemic, aleatoric, and total). The results are illustrated by configuration type contained within the dataset as well as the full dataset without separate consideration for configurations

Figure 7 shows the energy and force prediction performance of each of the models with respect to RMSE and RMSCE separated by structure type within the dataset. Although the model is evaluated independently on each of the different structures of the dataset, during training, all of the structures were shuffled together (i.e., the models were not trained with any knowledge of the human-labeled structure groups). Although there are not individual benchmarks for performance as a function of carbon structure, the original GAP-20 dataset paper [48] indicates in the supplementary material that the top performing atomic cluster expansion (ACE) model has an energy RMSE of 102 meV/atom and forces RMSE of 539 meV/Å. The GAP20 potential reports results of 1083 meV/atom and 725 meV/Å for energy and forces, respectively [48].

The top two panels of the figure show the performance with respect to energy RMSE and RMSCE. These results indicate that there is a very consistent performance across all model types and all configuration types on this large dataset. There is no single model that performs consistently the best with respect to energy RMSE across all structures. There are two outliers in the energy mean performance of interest: the ANN model for amorphous surfaces is significantly smaller than the other models for that configuration, and the MDN on graphene shows the single smallest RMSE of 7.2 meV/atom. The RMSCE similarly indicates that there is no clear winner across all configuration types, with each model type achieving the smallest or second smallest calibration errors on at least one of the carbon structures. The most notable outlier for energy calibration is again the MDN, but this time on the dimer structures, which results in 1.6 meV/atom RMSCE, representing near perfect uncertainty calibration.

These results are not mirrored when considering the performance of RMSE and RMSCE on the atomic forces, shown on the two bottom panels of Fig. 7. For mean predictions, the conclusions are largely the same, there is no single model with consistently best performing estimates of the forces across all structure types. Regarding individual structure performance, the ANN model achieves a very small RMSE on graphene. With respect to the force RMSCE, however, we see a break in the above trends. For force RMSCE, we observe that the calibration error for the deep ensemble of ANN-based NNPs consistently outperforms the other model types on all structures except for ‘amorphous bulk’ and “SACADA”, the two best-represented structures in the GAP-20 dataset. On these structures, the aleatoric and total uncertainty predictions provided by the MVE, MDN, and BNN all outperform the epistemic uncertainty provided by the deep ensemble.

**Fig. 8:**
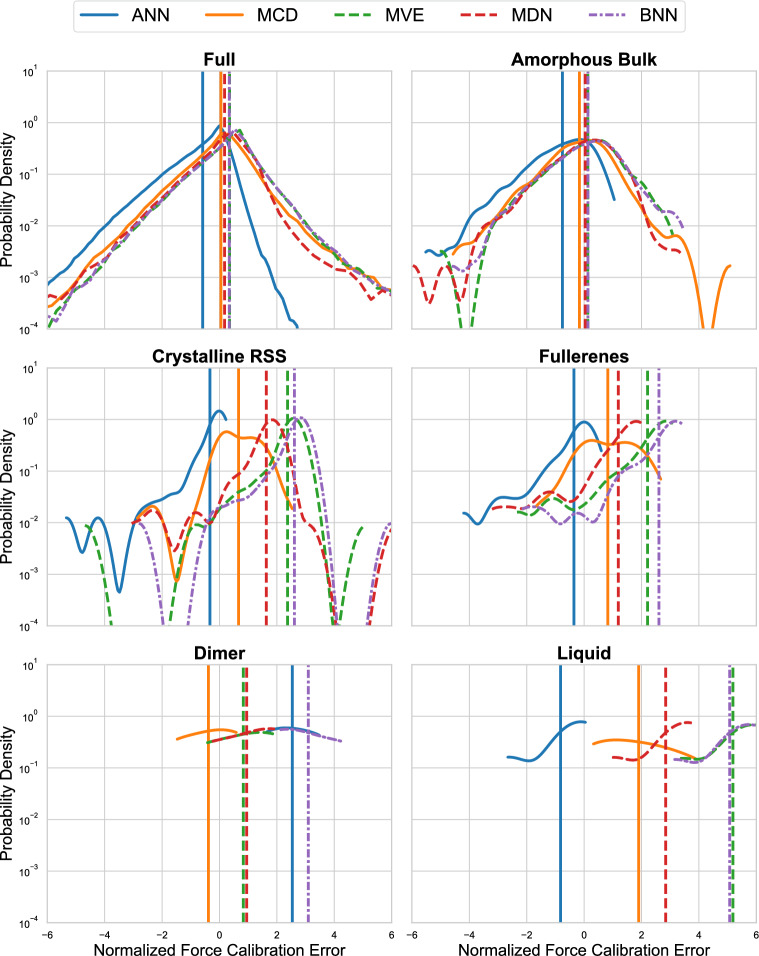
PDFs of the normalized force calibration error results on the test set for each of the different structures contained within the GAP-20 dataset. Calibration errors are separately provided on the full dataset for additional context

Figure 8 shows the individual PDFs for each of the five model’s force calibration error for the full test dataset (with all structures grouped together) and for five other structures chosen to highlight some general observations. The full PDF figure with each of the individual structures contained within the dataset is provided in the supplemental material. Similar to the performance on the MLEARN datasets, the ANN deep ensemble again consistently underestimates the true error associated with the prediction on the majority of the atomic structure labels. One main exception to this is the overprediction of uncertainty performance on the dimer sub-section of the data (shown in Fig. 8). In contrast to prior observations on the MLEARN datasets, the MCD models are not consistently the most overpredicting models with respect to the normalized force calibration, though still consistently overpredictive (except in the case of dimer), the models with native aleatoric uncertainty estimates are shown to have consistently higher normalized calibration errors than their epistemic counterparts. For certain particularly sparsely represented carbon structures (such as “liquid”), this behavior is particularly pronounced. In these less data-dense regions of the dataset, the performance of the ANN deep ensemble still tends to underpredict the true error, but the magnitude of the misalignment is, in many cases, significantly less severe than that of the aleatoric-based models. In these structures, we observe the epistemic uncertainty predictions to show much better calibration with true error and the aleatoric/total uncertainty estimates drastically overestimate the true error associated with the predictions. It is important to note that local ACSF descriptors may introduce category-dependent effects by representing certain atomic environments more effectively than others, particularly given the imbalanced training dataset density distribution of human-labeled GAP-20 categories. However, because the ACSF descriptors and data density splits are identical for all methods considered, the observed systematic differences in performance within a given structure category are attributable to method-dependent modeling differences (e.g., UQ method, loss function formulation, and hyperparameter choices).

**Fig. 9:**
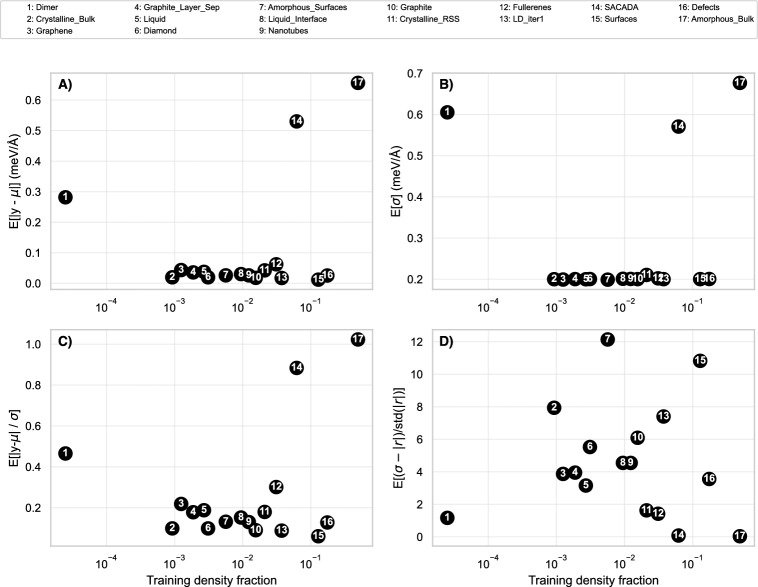
Impact of the data density on different quantities of interest: a) mean absolute error (MAE), b) mean standard deviation, c) mean MAE normalized by standard deviation, and d) the mean normalized calibration error for MDN evaluated on the GAP-20 dataset

To evaluate the impact of the training set density (Fig. 3) in more detail, Fig. 9 shows several different metrics of interest with respect to the training data density. Each panel of the plot shows the evaluation of the MDN model on the test portion of the GAP-20 dataset. The data shows that there is a general trend towards increasing normalized force calibration error as the training data density decreases, with the notable exception of dimer (as previously discussed). While these results agree with those of Fig. 8, they provide additional insight into the mechanism by which the UQ degradation occurs. The three best performing atomic structure types are amorphous bulk, SACADA, and Dimer; these atomic structures also display the highest MAE and highest mean standard deviation from the model outputs. This indicates that the model is accurately increasing standard deviation as the mean prediction errors increase. For the rest of the atomic structures, the MAE fluctuates about a relatively small value, meanwhile there is almost no corresponding fluctuations about the standard deviation. These standard deviation values are mostly uniform and the predicted error deviates from the actual model error. This suggests that a more robust strategy to prevent the standard deviation collapse in models with aleatoric uncertainty output layers could improve the accuracy of these methods in data-sparse regions.

In general, we find that the GAP-20 dataset provides a unique challenge for UQ-enabled NNPs. The wide range of data densities present in each of the human labeled structure types highlight the strengths and weaknesses of each of the methods studied. While the ANN ensemble methods showed consistent underestimation of the prediction error, they were far less sensitive to underrepresented regions of the dataset. The MCD model did not exhibit consistently poor performance as was identified with the MLEARN data, which suggests that this method may perform better with larger datasets. The aleatoric uncertainty estimates show very strong performance in general, but have high sensitivity to datasets that unevenly represent the input space and tend to significantly overpredict the errors in these regions. Finally, despite their high computational cost, we find that the total uncertainty estimate provided by the BNN does not consistently outperform or underperform the aleatoric uncertainty estimate provided by the single-shot methods.

### Hyperparameter Importance on UQ

The extensive hyperparameter tuning algorithm used an objective function set to minimize the validation loss of each model architecture. While the loss minimization is a standard objective for hyperparameter tuning, it is not guaranteed to directly translate to uncertainty quantification performance. Here we evaluate each of the top models from of the hyperparameter tuning runs that contain native UQ on the test set for energy and force RMSCE. After evaluating the RMSCE performance for each of the models, we train lightweight random forest regressors and perform importance permutation as implemented in the Scikit learn Python package. The full results for each of the hyperparameters are shown in the Supplementary Material.

**Fig. 10:**
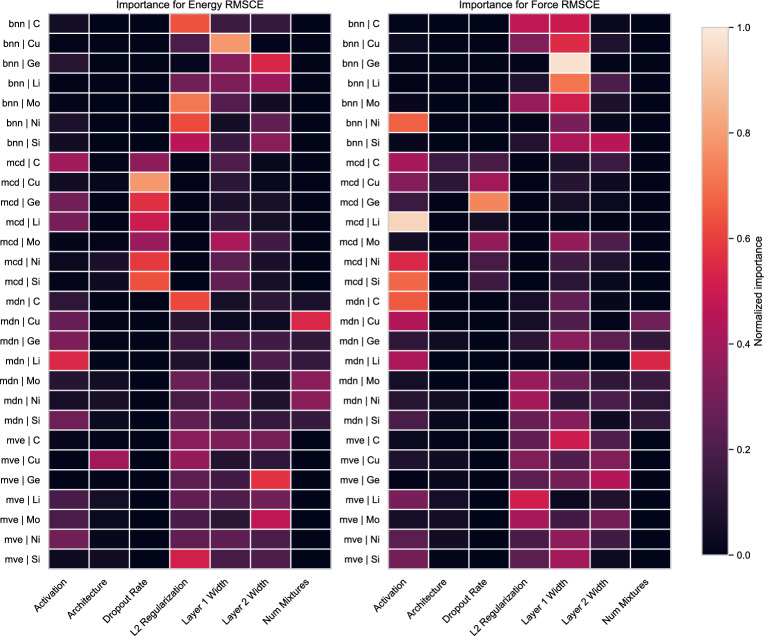
Heatmaps showing the importance of the various hyperparameters normalized by row. The left panel shows the importance of the hyperparameters for energy RMSCE and the right for force RMSCE

Figure 10 shows the results of the hyperparameter permutation importance analysis visualized as a heatmap normalized by the maximum importance value within each row. The left panel shows the importance of the hyperparameters for energy RMSCE and the right for force RMSCE. The results in each panel are sorted by model type to help with visual comparison and highlight important hyperparameters for each method type. Most of the models indicate a high importance attributed to layer width which shows that model capacity is an important factor for accurate UQ performance. It is important to note that this does not mean that increased model capacity is always beneficial, just that RMSCE is sensitive to model capacity and should always be tuned during potential development.

Sorting the results by model type allows easier identification of model specific trends. Explicit regularization is more important for MVE and MDN methods than BNN. The activation function is shown to be important for several of the datasets and model types, but has the strongest importance for MCD and MDN. Surprisingly, the dropout rate is the most important hyperparameter for only two of the MCD datasets germanium and copper, although it is a close second in the case of molybdenum. While most results show commonality among model type, the alternative grouping shown in the Supplementary Material helps identify trends specific to datasets independent of NNP method. Of particular interest is the importance of L2 regularization for all of the NNPs fit on the molybdenum dataset, particularly for the force RMSCE. Another interesting observation is the importance of the activation function for lithium, and nickel and silicon to a lesser degree. In sum, we find that the choice of hyperparameters can have a very large impact on the reliability of the uncertainty estimate; the first layer width is the most consistently important, but certain methods and datasets are more sensitive to other hyperparameters.

### Evaluation time

One of the key disadvantages of ensemble-based methods is the increase in evaluation cost associated with computing predictions from the entire ensemble before acquiring an uncertainty estimate. On the other hand, single-shot approaches can provide uncertainty estimates with a single forward pass through the network. While the additional number of parameters associated with the single-shot methods will tend to increase their computational cost with respect to a single evaluation of a simple ANN, we find those cost increases to be negligible with respect to the cost of the ensemble evaluations.

**Fig. 11:**
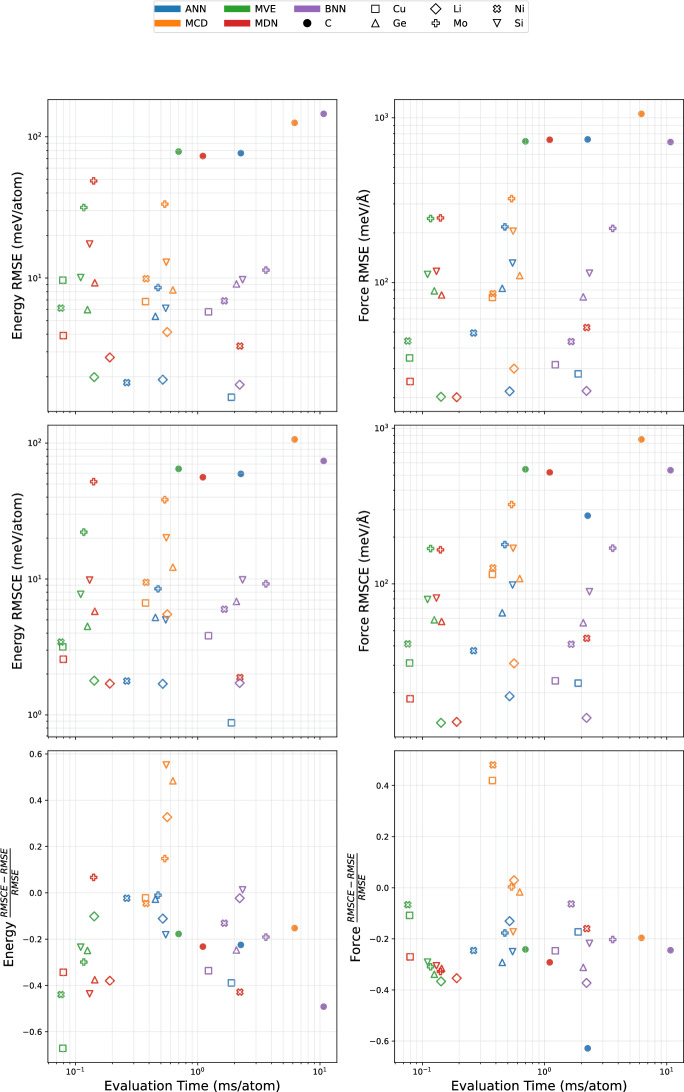
Comparison of the evaluation times and RMS performance for each of the different model types on a dual socket Intel Xeon Sapphire Rapids CPU

Figure 11 shows the comparative RMS performance and timing estimates for each of the different model types during evaluation on each of the seven datasets. These timing estimates were recorded on a single node using a Dual socket Intel Xeon Sapphire Rapids CPU. A CPU was used for these measurements since there was no noted increase in computation speed compared to an NVIDIA H100 GPU, due to the relatively small number of parameters required for each of these NNPs. The ANN deep ensemble consists of 10 unique model evaluations, the MCD and BNN models were each evaluated 10 times to compute the epistemic uncertainty, and the MVE and MDN were each evaluated a single time. The performance results on the y-axes are the same as previously shown in Figs. 5 and 7. On a per evaluation basis, the ANN is the fastest, so if these ensemble evaluations are appropriately parallelized, the evaluation wall time can be decreased by a factor of 10, however, this still represents an increase in the overall number of computational resources required. With these caveats, we present the computational time per atom as an approximate representation of the overall computation cost for these methods.

The first two panels show the energy and force RMSE and RMSCE, respectively. The third and final panel in Fig. 11 shows the normalized difference between the RMSCE and RMSE which is indicative of the relative performance of each of the models for energy and forces. For models with no UQ, the RMSCE would be identical to the RMSE. Therefore, the normalized difference between these quantities serves as a useful benchmark to quantify the relative improvement of explicit UQ over an uncertainty unaware model, with lower values indicating better performance.

The data show that there is no consistently “best” model with respect to all aspects of performance and computational cost. The results from the MLEARN datasets are presented for each element as different hollow markers, colored by NNP method. These points collectively indicate that the ANN deep ensemble is able to provide highly competitive RMSE and RMSCE (particularly for total energy predictions), but there is usually a single-shot method that provides similar performance with an order of magnitude cost reduction per atom. While that trend is consistent for the RMSE predictions on the GAP-20 dataset, the aforementioned overprediction of uncertainties from the single-shot methods on underrepresented portions of the dataset leads to 3–5X inferior RMSCE performance on the full GAP-20 dataset. Therefore the optimal choice of UQ method for NNPs is highly dependent upon resource constraints, the characteristics of the underlying dataset, and the intended application. We note that while the BNNs are significantly more computationally expensive to train and evaluate, their performance is not proportionally better than the alternative methods.

## Discussion

While NNPs have long been shown to deliver superior performance when compared to traditional IAPs, obtaining well-calibrated and consistent UQ has been a challenge. In this study, we examined the performance of five different methods for obtaining uncertainty estimates from NNPs: a deep ensemble of ANNs, multiple evaluations of a single MCD network, aleatoric predictions from single-shot MVE and MDN networks, and total uncertainty predictions from a BNN. To properly identify and evaluate the best performing models of each type, we developed a framework combining KLIFF and PT-MELT, which allowed for efficient model flexibility and automated hyperparameter tuning using the Ray Tune package. This robust hyperparameter tuning not only provided a means to ensure high-quality results from each model type, but also a native way to form accurate deep ensembles for epistemic uncertainty estimates.

Through the course of this study, we found hyperparameter tuning to be crucial, with some methods and datasets more sensitive to hyperparameters than others. This sensitivity to hyperparameters as a function of method and dataset is difficult to predict *a priori*; neglecting to perform individual hyperparameter tuning routines on each combination is likely to result in comparisons between sub-optimal models. Such comparisons can lead to over-generalized conclusions about a method’s efficacy which may just be an artifact of poor model parameters. Even with a robust hyperparameter tuning workflow, as implemented in this study, there is no guarantee the optimal set of hyperparameters is discovered. In particular, we note in this study that the MCD methods tend to consistently underperform the other methods evaluated. This could be a product of a poor method, or it could be that the optimal hyperparameters exist outside of the ranges explored (Table 2).

Across all datasets, we found that while the epistemic uncertainty provided by a deep ensemble of NNPs achieves a low RMSCE, the individual uncertainty estimates consistently underpredict the true error. The aleatoric uncertainty estimate provided by single-shot models can achieve equally low RMSCE and do not consistently underpredict the true error in regions of the dataset that are well represented. However, in regions of the dataset that are underrepresented (as is the case in certain GAP-20 labels), the aleatoric uncertainty tends to drastically overpredict the error. Additionally, our results suggest that the MCD method is highly dependent on the total amount of data available for training (with good performance on larger datasets, and poor performance on smaller datasets). Unfortunately, the BNN method did not consistently outperform the other methods, which is disappointing considering the substantial increase in computation cost associated with training and evaluating these models. For these more computationally intensive methods, a much higher degree of performance would be required to justify their widespread use outside of academic settings.

Overall, we found that no method consistently outperformed the others on all metrics, especially when computational cost is considered. We support the conclusion of the prior work of Tan et al. [28] that single-shot methods do not consistently outperform ensemble-based approaches on all metrics. However, we also claim that the ensemble-based approaches examined here do not consistently outperform single-shot methods. Each UQ method has performance trade-offs that need to be considered within the context of how they are intended to be deployed. Until a method is found that always outperforms every other method on any arbitrary dataset, it is left to the practitioner to determine which performance metrics are more important for their intended application.

## Conclusions

In conclusion, we find that there is not a single best method for jointly providing accurate energy/force predictions and well-calibrated uncertainty estimates. We show that single-shot methods are capable of delivering competitive UQ on both energy and forces in regions where there is sufficiently dense data. In regions of the data space that are inadequately sampled (as is often the case in certain active-learning loops), the single-shot methods consistently estimated larger uncertainty than the true model error. While the epistemic uncertainty approximation from deep ensembles remains the standard due to its simplicity, they tend to provide an overall underestimation of the true error, albeit with less degree of sensitivity to data irregularities than their single-shot aleatoric counterparts. Given the significant computational savings associated with single-shot approaches and their competitive performance, these single-shot methods should continue to be benchmarked to determine if these characteristics hold across a wider range of datasets and how they can be leveraged in production environments.

## Supplementary Information

Additional file 1.

## Data Availability

The MLEARN dataset is available courtesy of the original authors here [44]. The GAP-20 dataset will be available within the Orchestrator data module at a future date [50].
