# Supplementary material for: Benchmarking the performance of uncertainty quantification methods for neural network-based interatomic potentials
Source: J Cheminform. 2026 Apr 13;18:67. doi: 10.1186/s13321-026-01193-7 (PMC13188420; doi:10.1186/s13321-026-01193-7)
Supplement: Supplementary file 1 — Additional file 1. [file 13321_2026_1193_MOESM1_ESM.pdf]

Benchmarking the Performance of Uncertainty Quantification Methods for Neural Network-based Interatomic Potentials: **Supplementary material**

Nicholas T. Wimer[1*], Juliane Mueller[1], Sebastien Hamel[2], Vincenzo Lordi[2*]

[1]National Renewable Energy Laboratory.
[2]Lawrence Livermore National Laboratory.

*Corresponding author(s). E-mail(s): nwimer@nrel.gov; lordi2@llnl.gov;
Contributing authors: juliane.mueller@nrel.gov; hamel2@llnl.gov;

1 Introduction

This supplementary material document follows the organization of the main manuscript and aims to provide additional useful information throughout. The information contained within this document is purely supplementary and is not required to understand the methods, experiments, results, or conclusions in the main manuscript.

2 Description of Datasets

Complementary description of the MLEARN and GAP-20 datasets examining the total number of atoms contained within each portion of the datasets. In the main manuscript we present the data distribution in terms of total number of atomic configurations. Here, Supplementary Figure S1 shows the same distribution of the train, validation, and test subsets for each dataset in terms of the total number of atoms contained within each of the subsets.

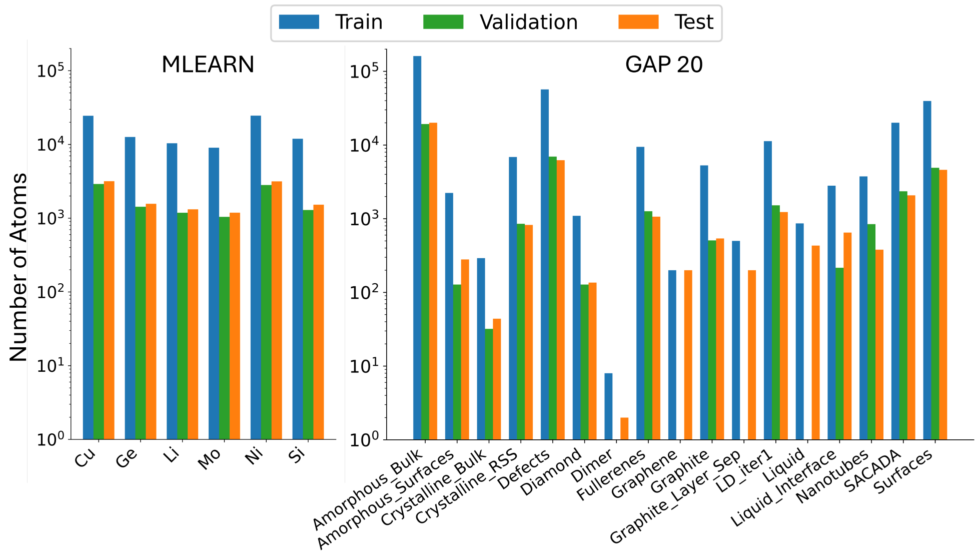

Supplementary Figure S1: Distribution of the total number of atoms contained within the training, validation, and test splits for each of the different datasets.

3 Hyperparameter Tuning

Results from the top 25% of the hyperparameter tuning models from the copper MLEARN dataset. The data shown in Supplementary Figure S2 and S3 are from the same hyperparameter tuning run shown in Figure 4 in the main document. Supplementary Figure S2 shows the best score achieved during hyperparameter tuning as a function of activation function, block 1 width, block 2 width, and L2 regularization. Each point in the scatter plot is a different model that was trained. Supplementary Figure S3 shows the hyperparameter correlation matrix indicating the full cross-correlation between all the hyperparameters included in this tuning run and the best score achieved during the tuning sweep.

Supplementary results from the hyperparameter importance study in the main text are presented here. Figures S4 – S17 show the uncertainty quantification (UQ) performance on force root mean square calibration error (RMSCE) for each of the top models from the hyperparameter turning runs. The results are shown with respect to each of the different hyperparameter choices that were modified during the tuning runs. The importance of each of these hyperparameters are evaluated and shown in the main manuscript text.

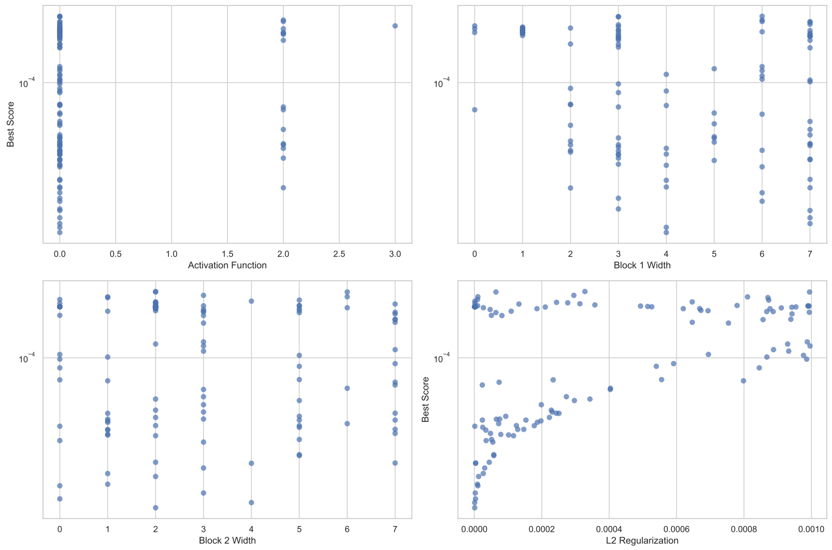

Supplementary Figure S2: Best validation loss scores achieved for each of the top 25% of models during the hyperparameter tuning run as a function of hyperparameter. Each point on the scatter plots represents a trained model with points lower on the y-axis representing the best performing models.

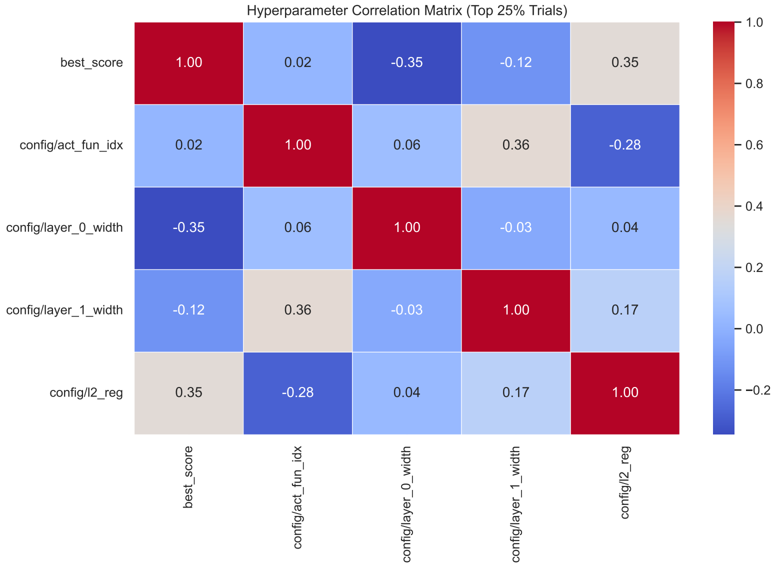

Supplementary Figure S3: Correlation matrix of the different hyperparameters and the best validation loss score achieved for the top 25% of trained models.

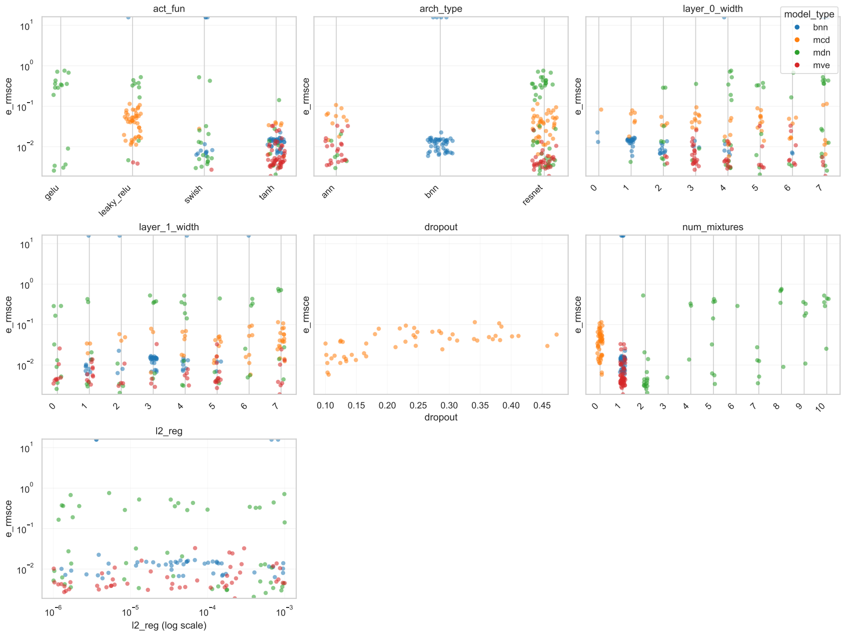

Supplementary Figure S4: Energy RMSCE results for the top models from the Cu MLEARN dataset.

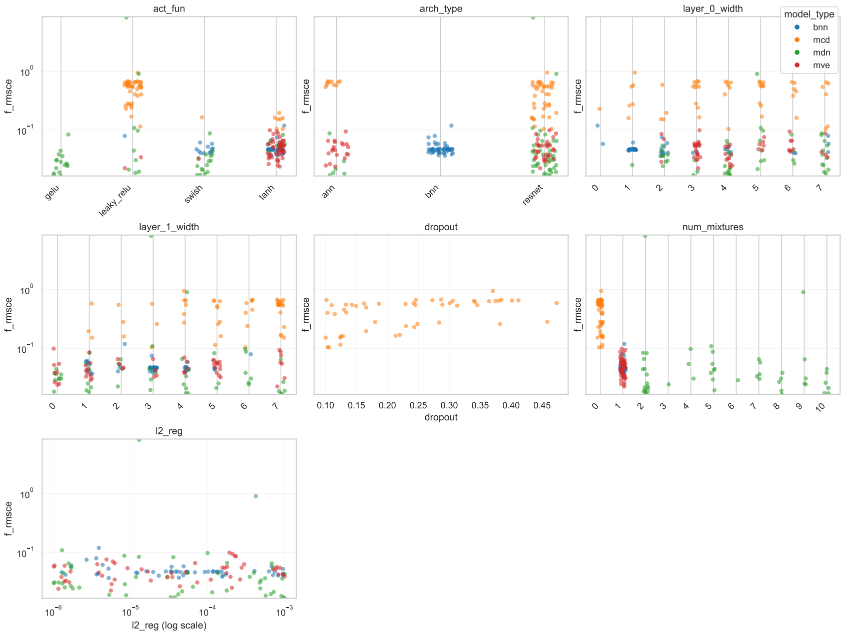

Supplementary Figure S5: Force RMSCE results for the top models from the Cu MLEARN dataset.

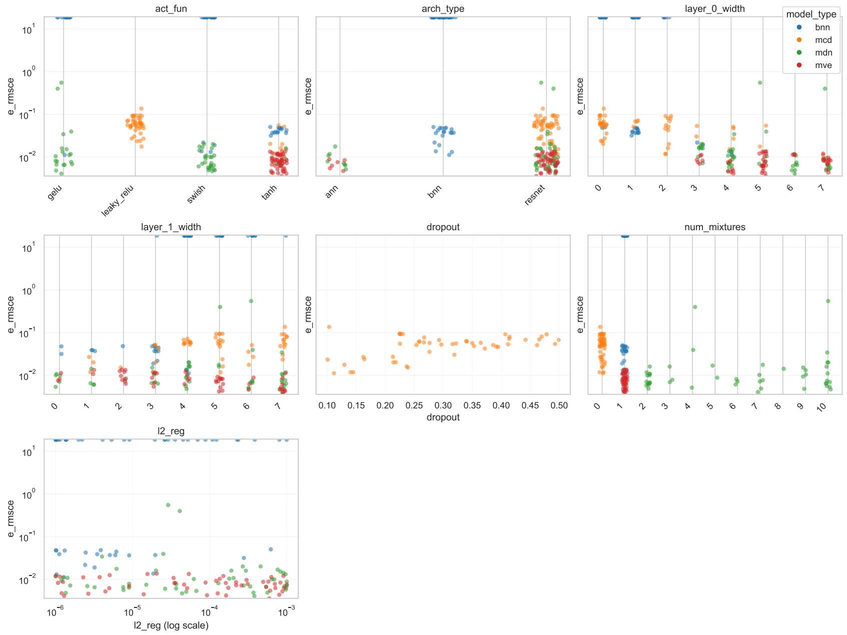

Supplementary Figure S6: Energy RMSCE results for the top models from the Ge MLEARN dataset.

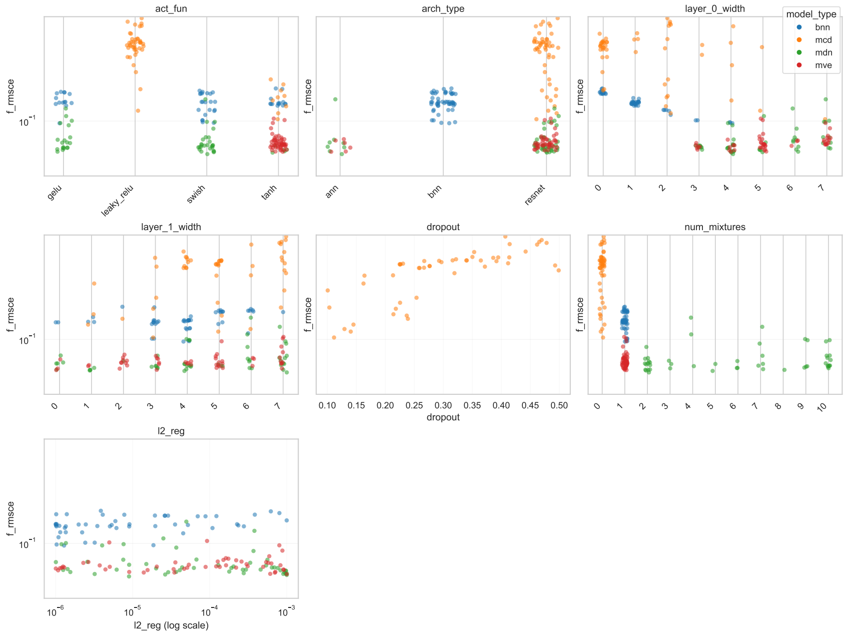

Supplementary Figure S7: Force RMSCE results for the top models from the Ge MLEARN dataset.

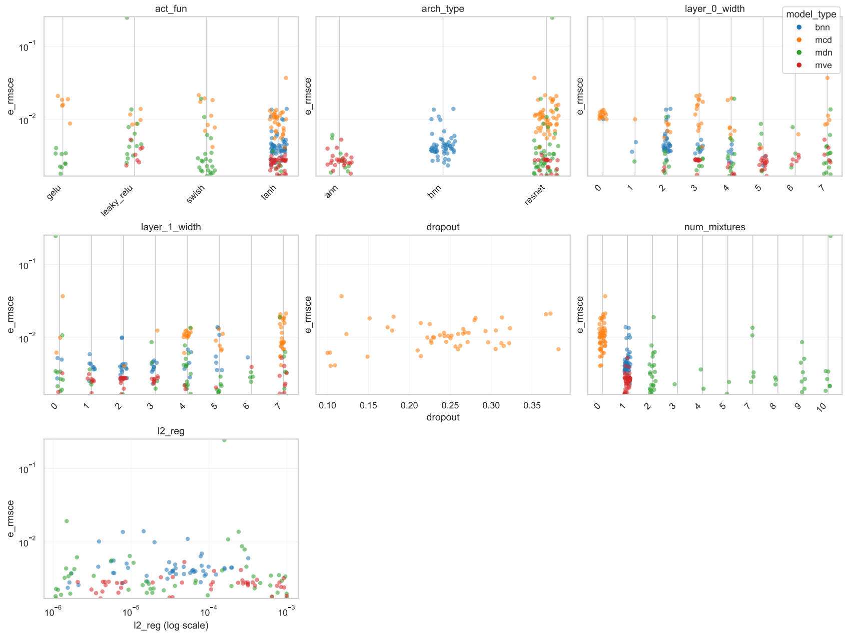

Supplementary Figure S8: Energy RMSCE results for the top models from the Li MLEARN dataset.

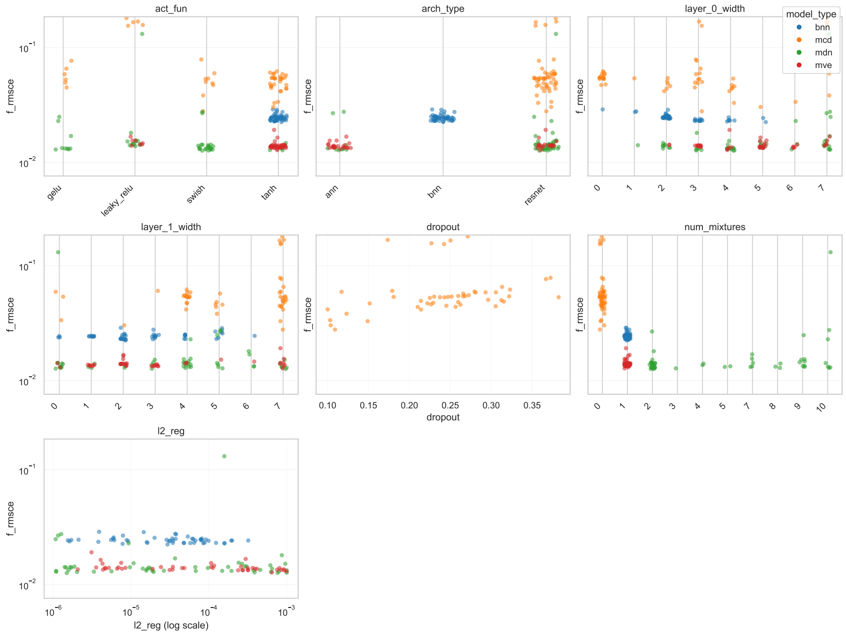

Supplementary Figure S9: Force RMSCE results for the top models from the Li MLEARN dataset.

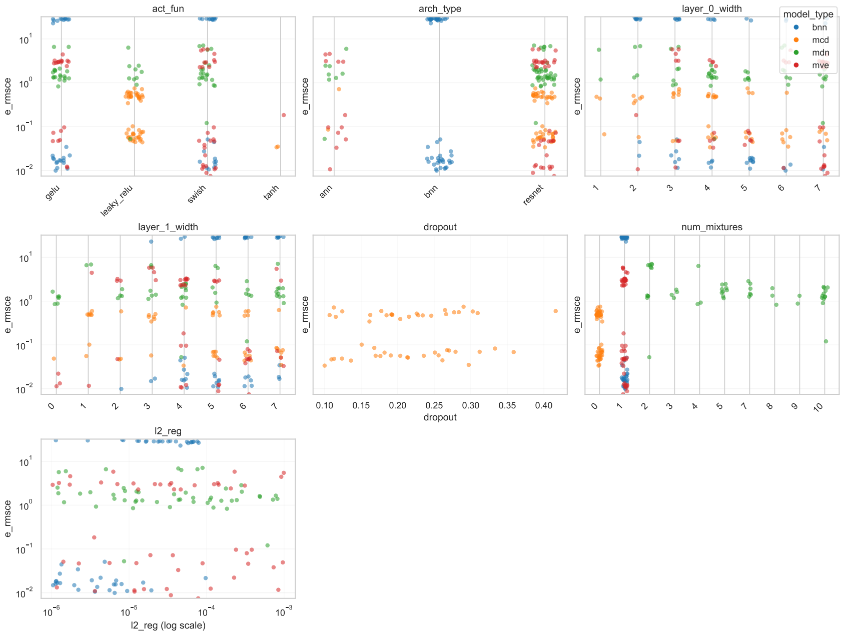

Supplementary Figure S10: Energy RMSCE results for the top models from the Mo MLEARN dataset.

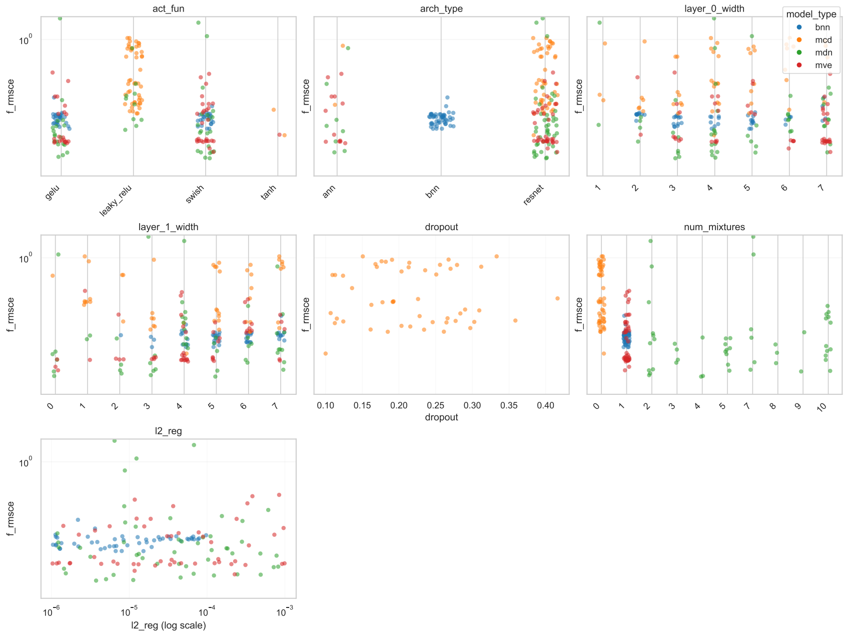

Supplementary Figure S11: Force RMSCE results for the top models from the Mo MLEARN dataset.

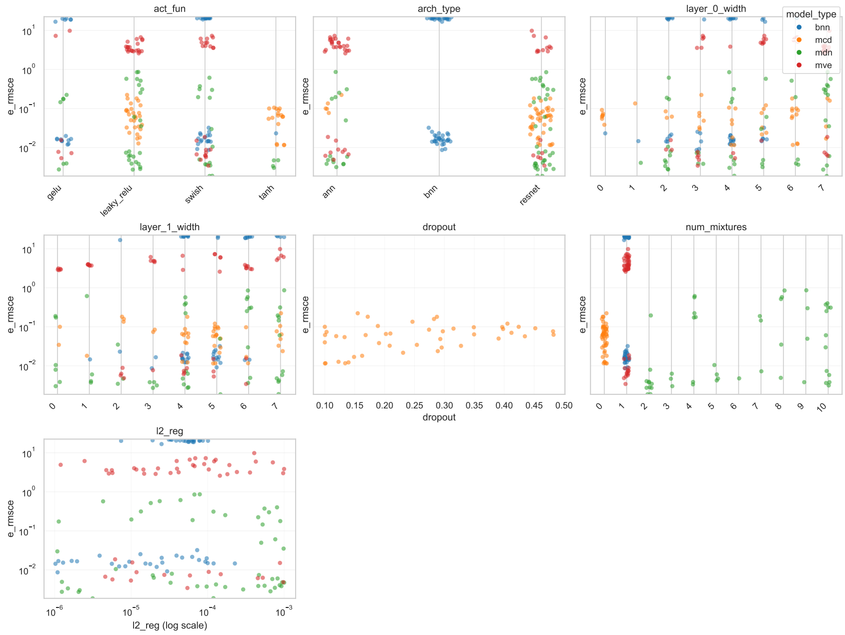

Supplementary Figure S12: Energy RMSCE results for the top models from the Ni MLEARN dataset.

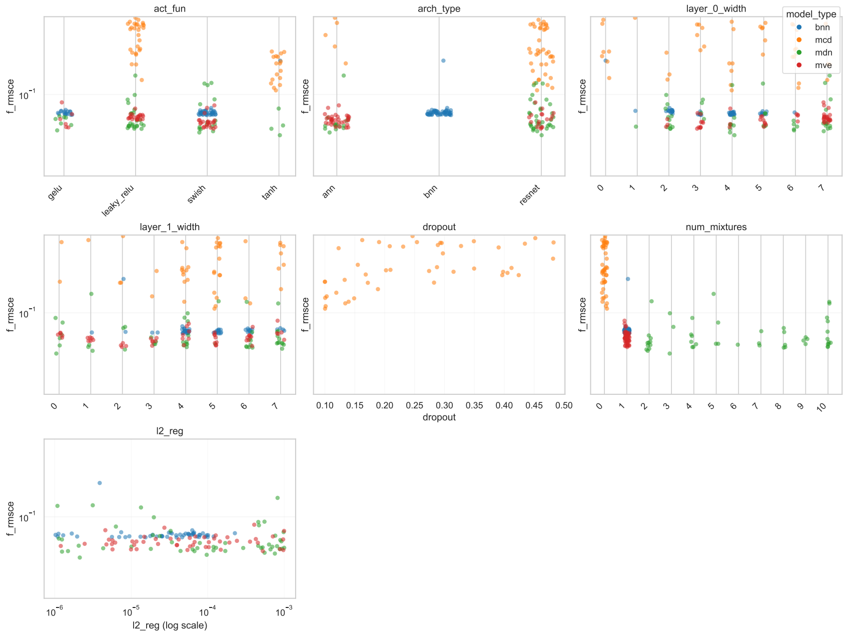

Supplementary Figure S13: Force RMSCE results for the top models from the Ni MLEARN dataset.

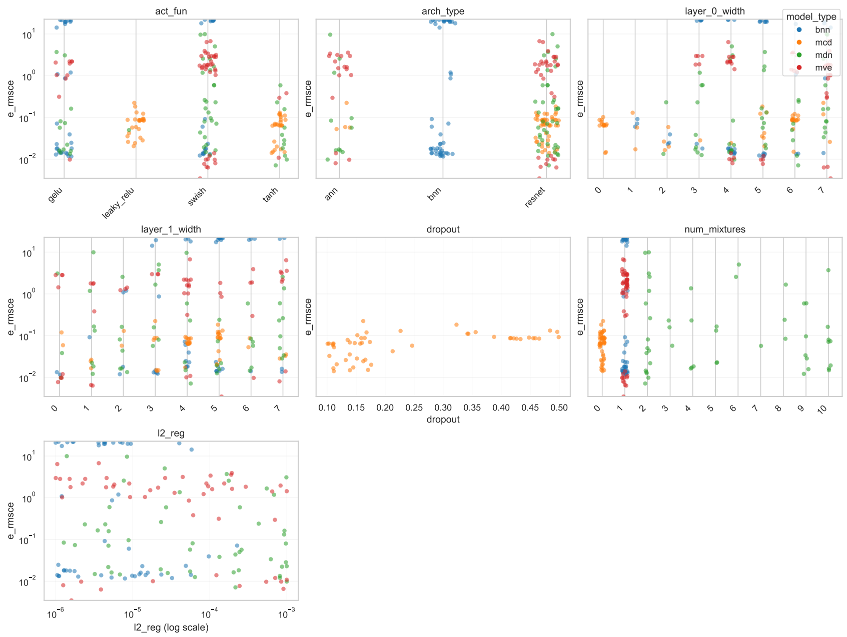

Supplementary Figure S14: Energy RMSCE results for the top models from the Si MLEARN dataset.

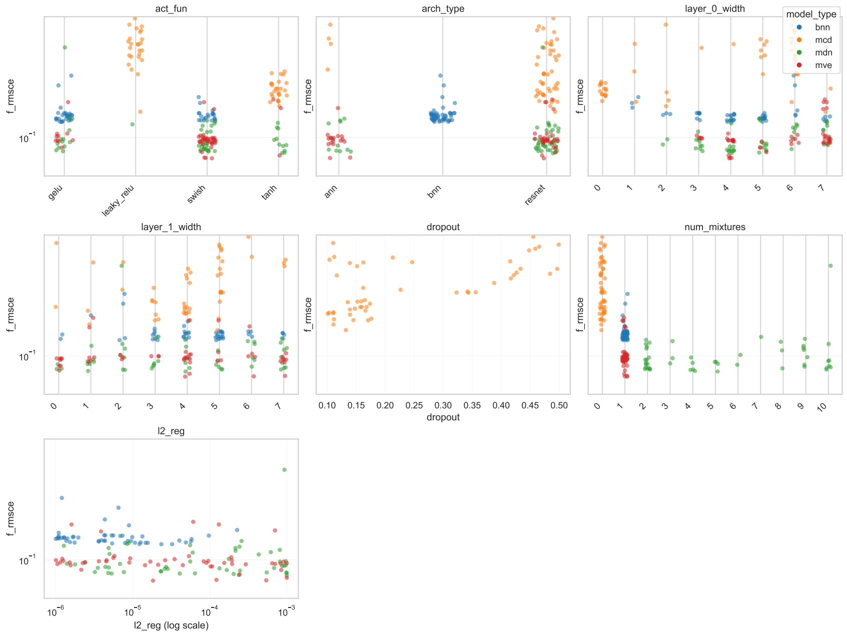

Supplementary Figure S15: Force RMSCE results for the top models from the Si MLEARN dataset.

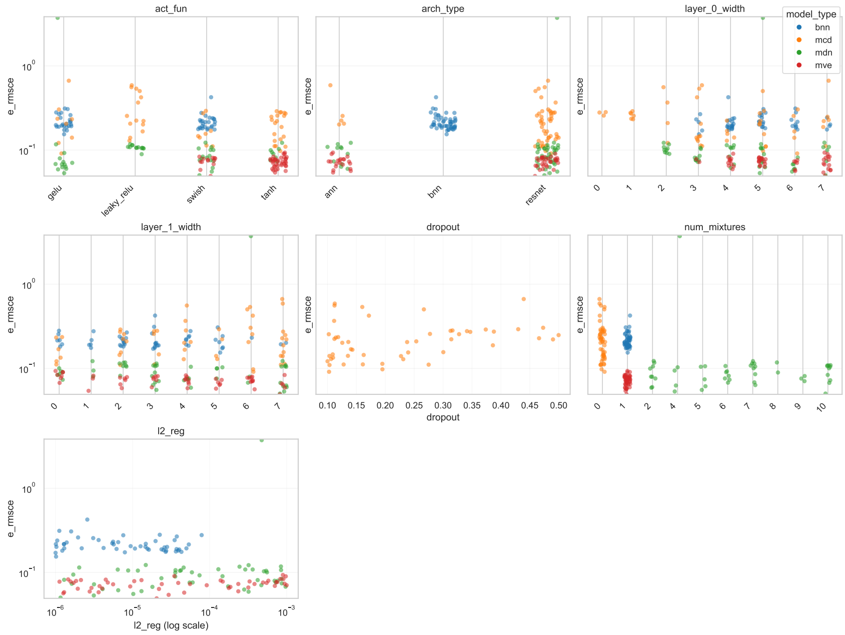

Supplementary Figure S16: Energy RMSCE results for the top models from the C GAP-20 dataset.

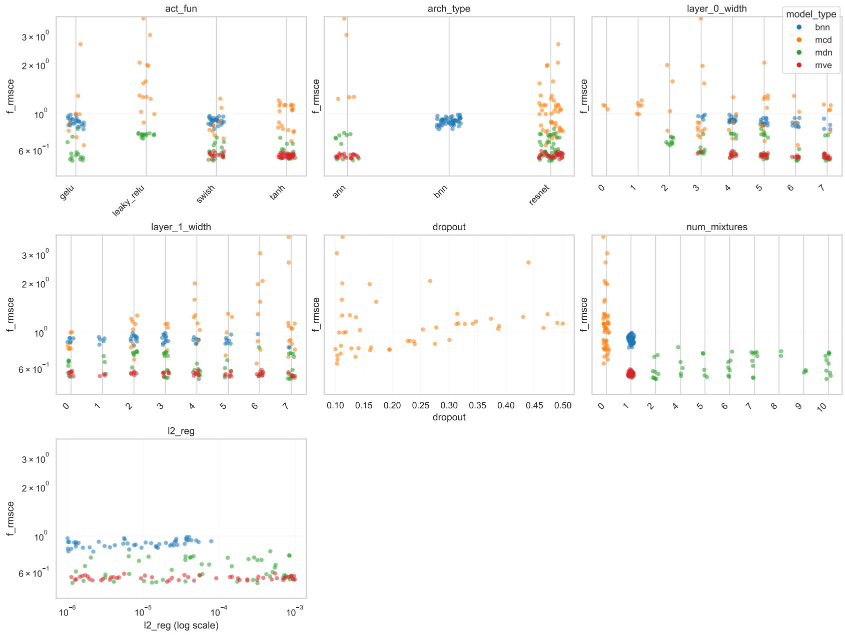

Supplementary Figure S17: Force RMSCE results for the top models from the C GAP-20 dataset.

3.1 Impact of Ensemble size

In the main manuscript text, we discussed the impact of ensemble size on the root mean square error (RMSE) and RMSCE performance of a mean-variance estimation (MVE) model. Here we show all of the ensemble performances for each of the top 20 models on the same Cu portion of the dataset as a point of comparison. RMSCE results are only available for models with native UQ. Supplementary Figure S18 shows the ensemble results for the artificial neural network (ANN), Supplementary Figure S19 the ensemble results for Monte Carlo dropout (MCD), Supplementary Figure S20 the ensemble results for MVE, Supplementary Figure S21 the ensemble results for mixture density network (MDN), and Supplementary Figure S22 the ensemble results for Bayesian neural network (BNN). For each of the ensembles, the ensemble mean is not able to outperform the single best model, except for the BNN RMSCE for forces (Supplementary Figure S22).

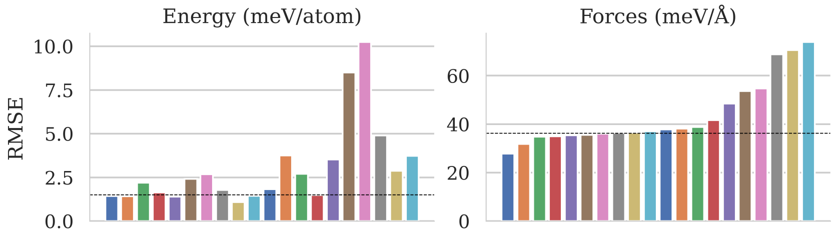

Supplementary Figure S18: Performance of the top 20 artificial neural network (ANN) models trained on the copper MLEARN dataset.

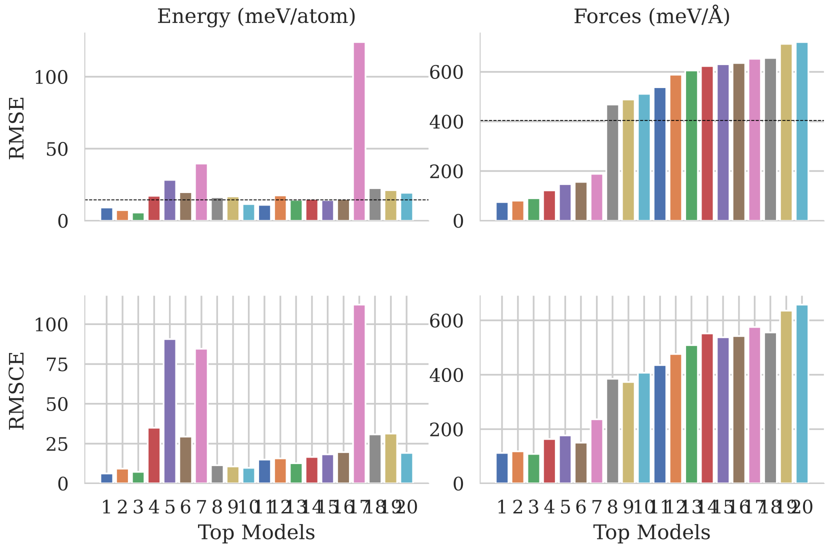

Supplementary Figure S19: Performance of the top 20 Monte Carlo dropout (MCD) models trained on the copper MLEARN dataset.

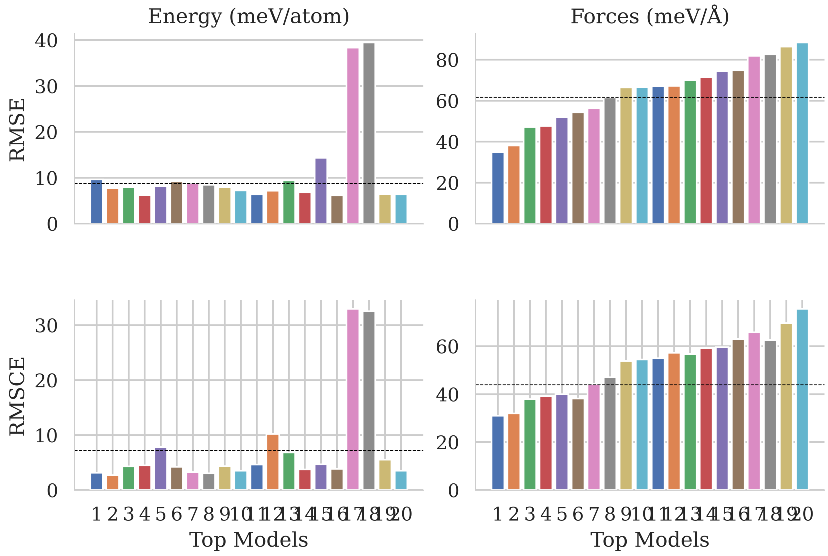

Supplementary Figure S20: Performance of the top 20 mean-variance estimation (MVE) models trained on the copper MLEARN dataset.

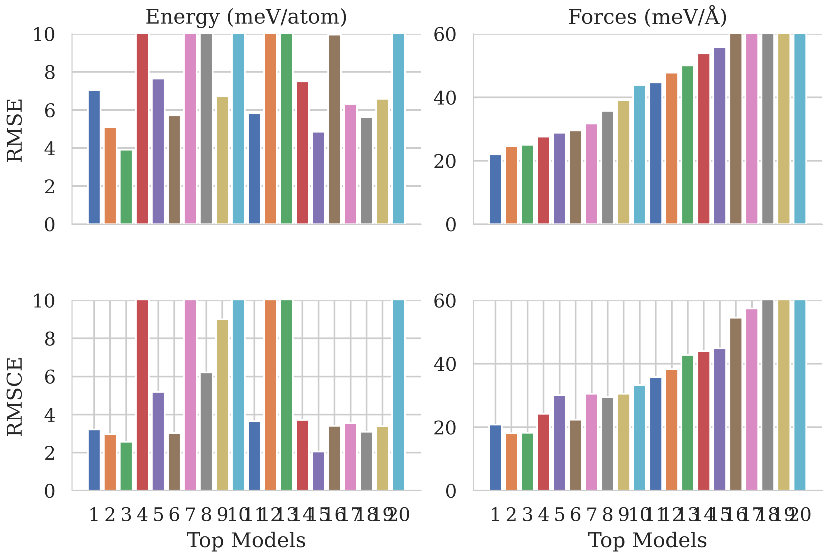

Supplementary Figure S21: Performance of the top 20 mixture density network (MDN) models trained on the copper MLEARN dataset.

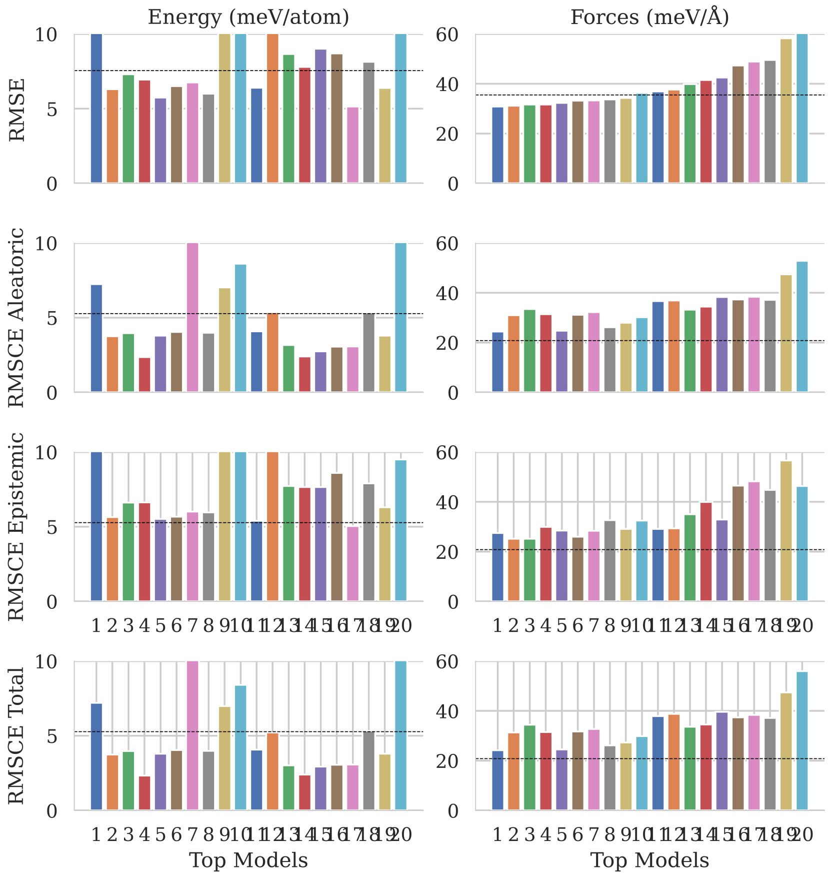

Supplementary Figure S22: Performance of the top 20 Bayesian neural network (BNN) models trained on the copper MLEARN dataset.

4 Full PDF Plot for GAP-20

In the manuscript we show a subset of the GAP-20 dataset due to space limitations. Here we present the full set of PDFs. Supplementary Figure S23 shows the full probability density function (PDF) for each of the human labelled structure divisions from the GAP-20 dataset with figure axes that are large enough to show the long tailed outliers that exist in some of the divisions. In the manuscript, we narrowed the range of the plots to highlight the shape of the PDFs and representative behavior of the full data.

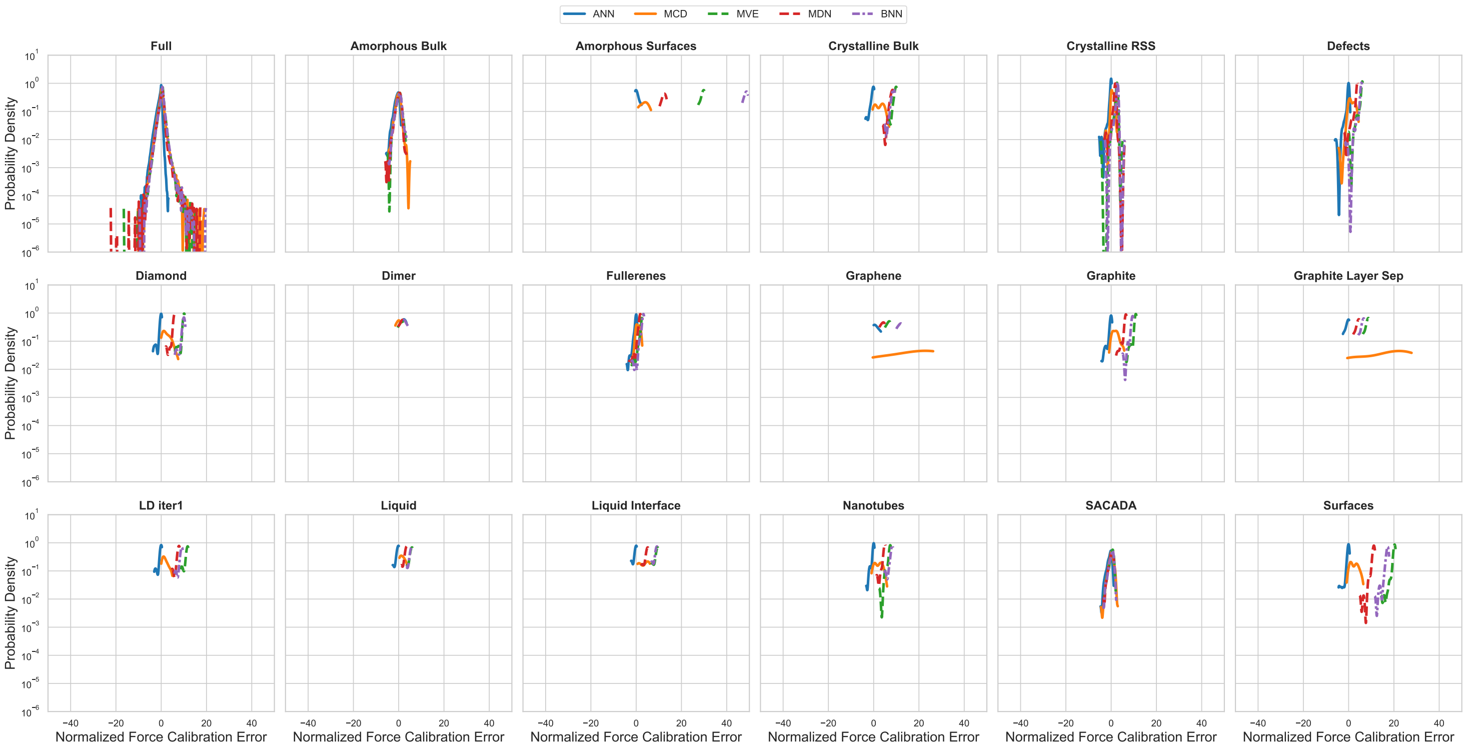

Supplementary Figure S23: PDFs of the normalized force calibration error results on the test set for each of the different structures contained within the GAP-20 dataset. Calibration errors are separately provided on the full dataset for additional context.

5 Analysis of Data Density on UQ Performance

Here we provide the full results from the data density analysis on the UQ performance for energy and force RMSCE. Figures S24 – Figures S33 show the same plots displayed in the main manuscript, but for all NNP model types: ANN, MCD, MVE, MDN, and BNN. The results are shown for every subset of the GAP-20 dataset. The results show that better RMSCE performance is correlated with models that do not collapse to a constant uncertainty quantification across the atomic structure subsets.

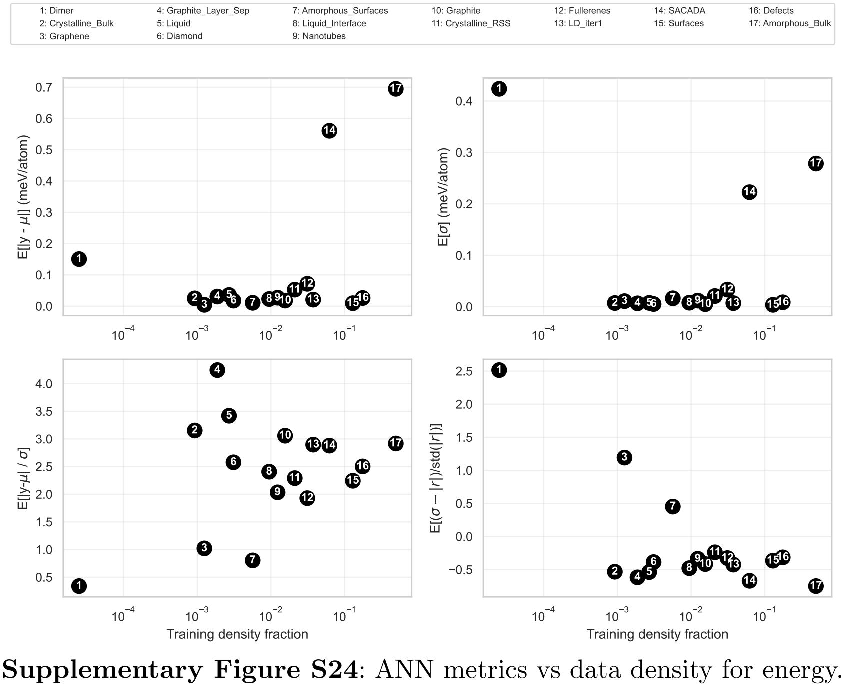

Supplementary Figure S24: ANN metrics vs data density for energy.

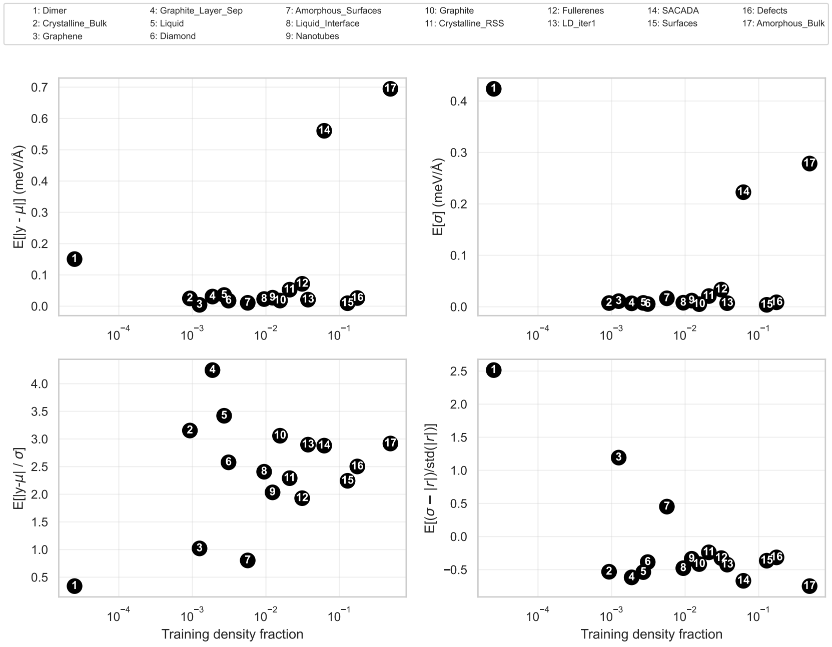

Supplementary Figure S25: ANN metrics vs data density for forces.

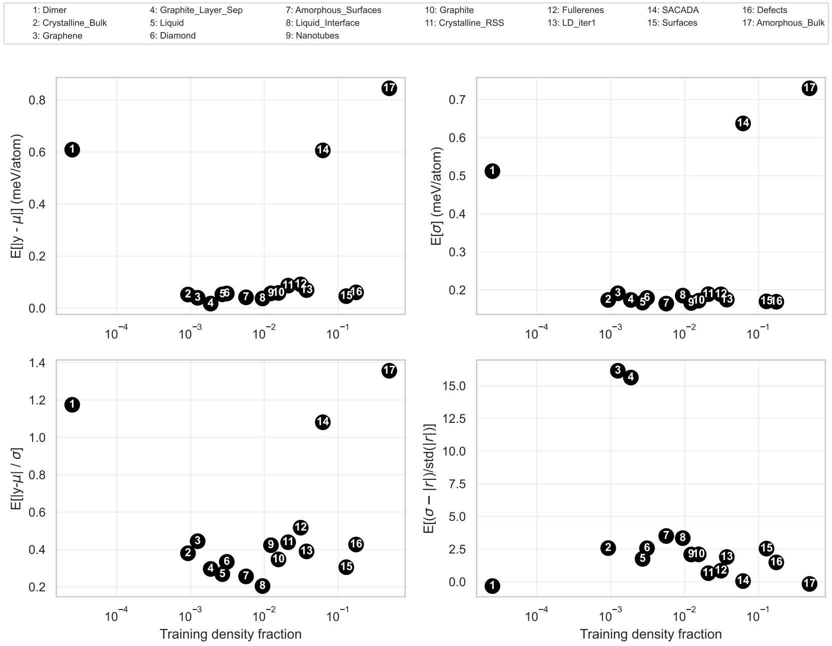

Supplementary Figure S26: MCD metrics vs data density for energy.

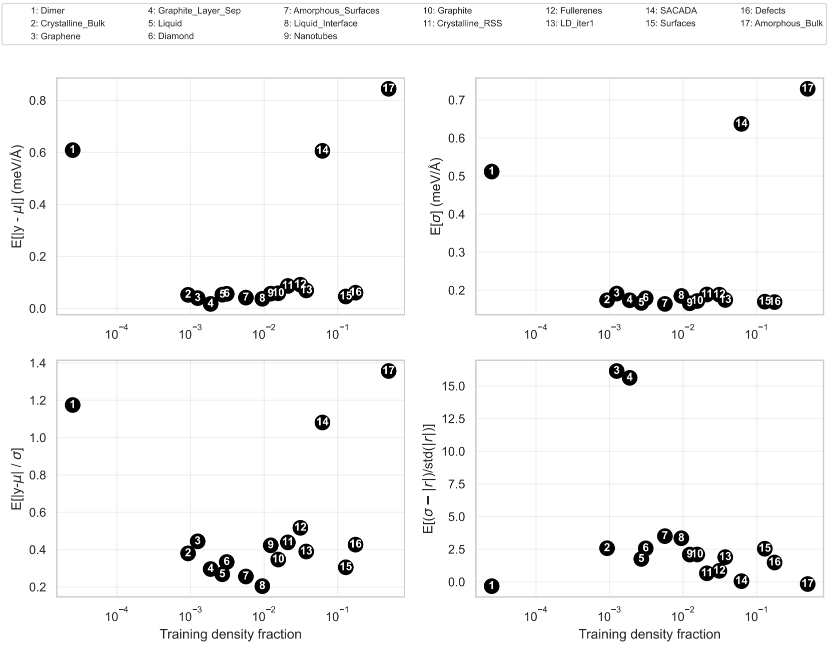

Supplementary Figure S27: MCD metrics vs data density for forces.

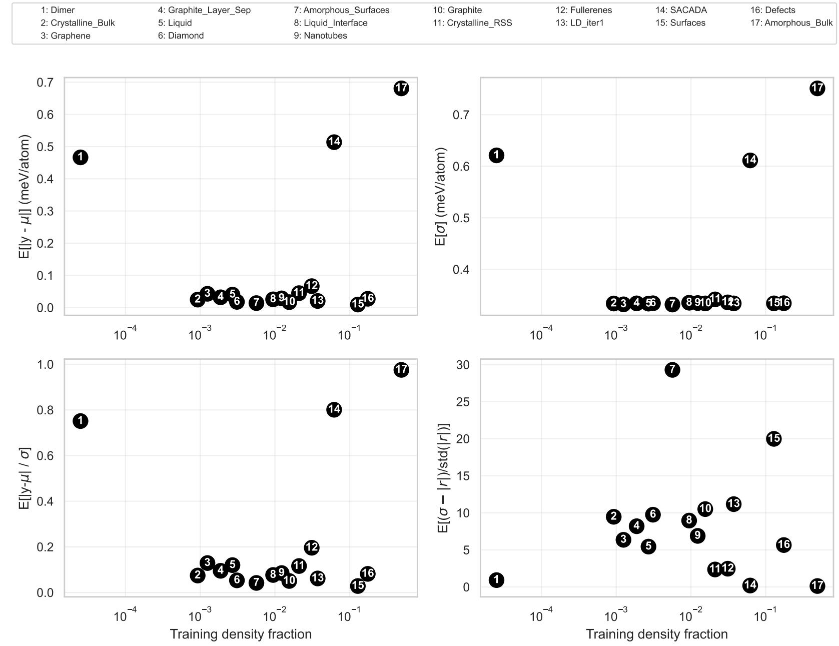

Supplementary Figure S28: MVE metrics vs data density for energy.

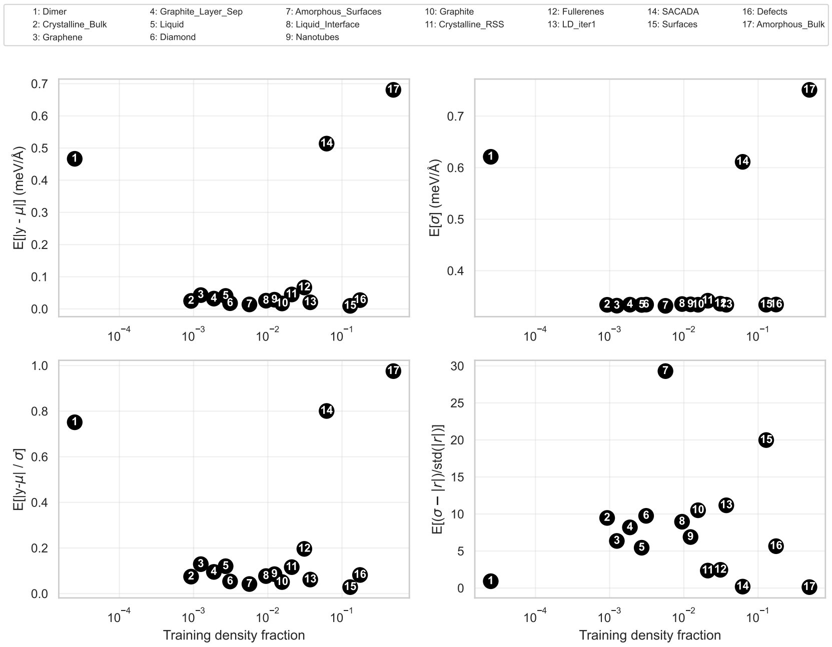

Supplementary Figure S29: MVE metrics vs data density for forces.

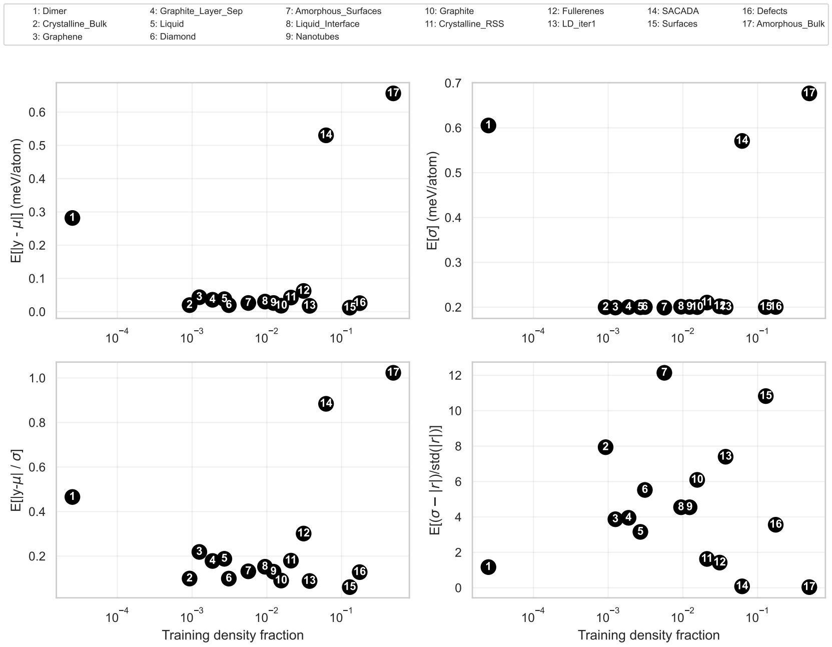

Supplementary Figure S30: MDN metrics vs data density for energy.

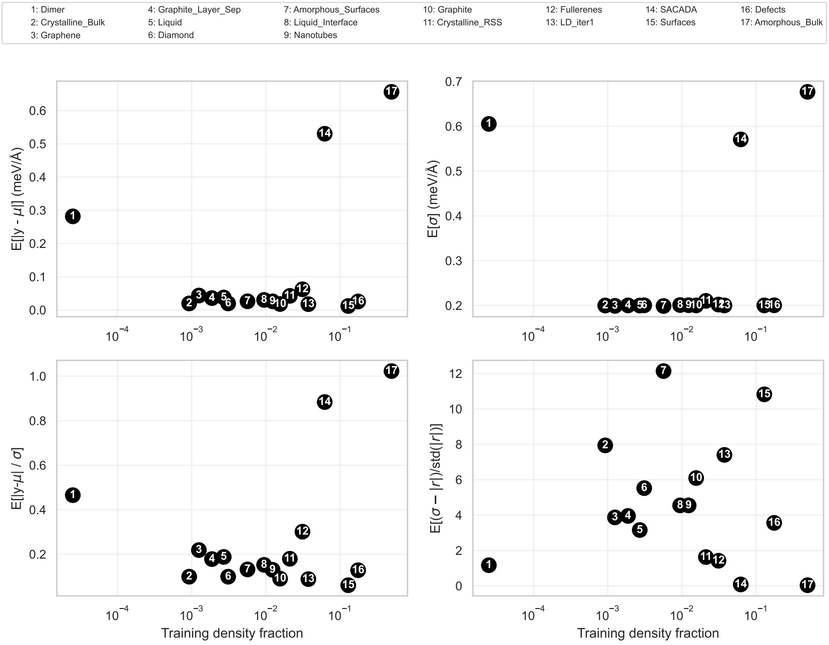

Supplementary Figure S31: MDN metrics vs data density for forces.

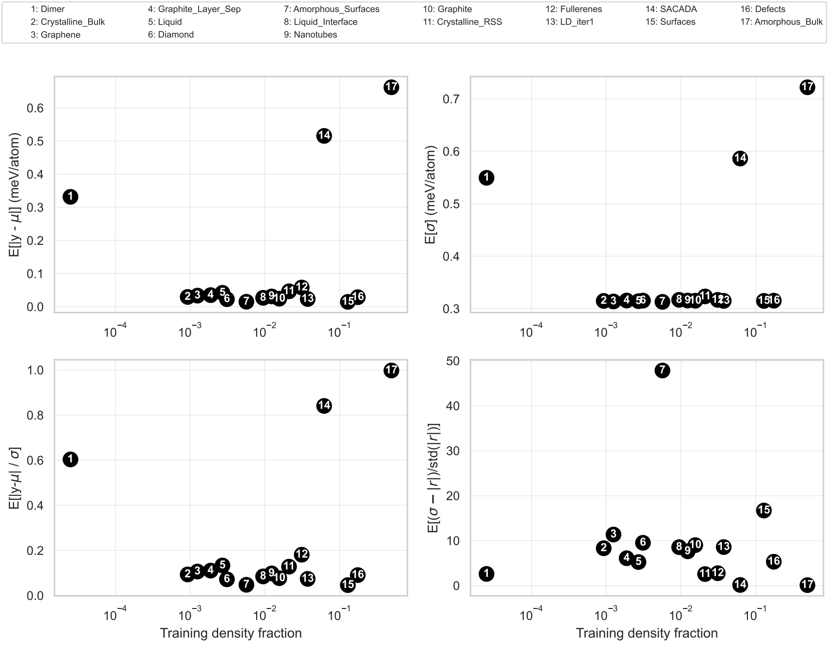

Supplementary Figure S32: BNN metrics vs data density for energy.

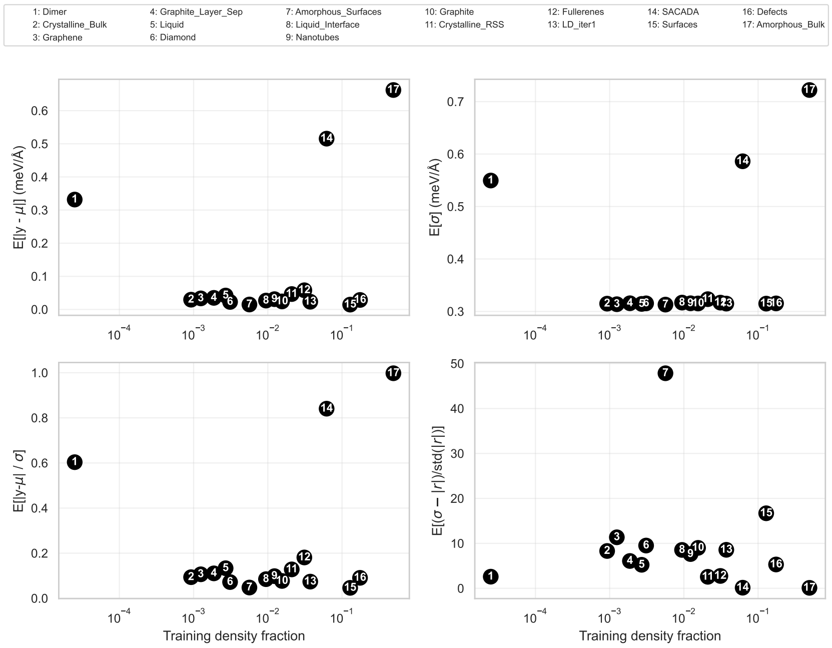

Supplementary Figure S33: BNN metrics vs data density for forces.

6 Alternative Visualizations of Model Performance

In the main manuscript, performance was shown in the form of bar and PDF plots. Here we present the full predictive performance for each atomic structure in the form of parity plots, colored by the associated uncertainty, shown in Supplementary Figure S34 – Supplementary Figure S68. The legend of each plot contains the RMSE, the prediction interval coverage probability (PICP), and mean prediction interval width (MPIW).

6.1 Cu MLEARN

Supplementary Figure S34 – Supplementary Figure S38 show the energy and forces parity plots for all model types trained on the copper MLEARN dataset.

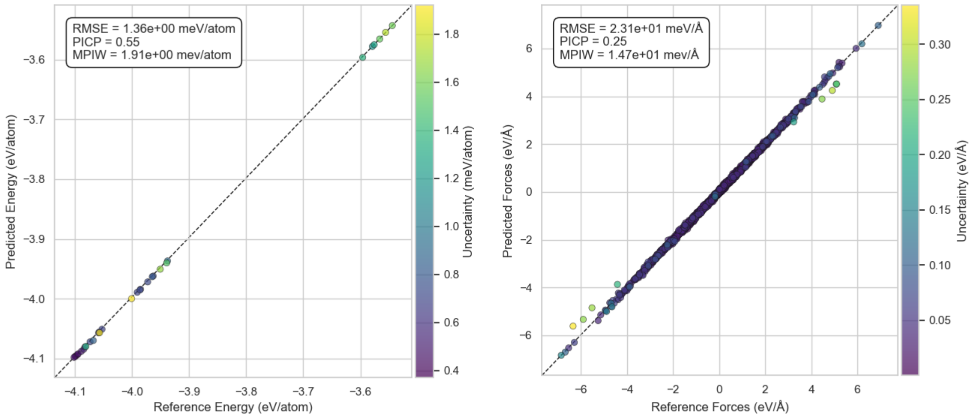

(a) Energy predictions on the test dataset. (b) Force predictions on the test dataset.

Supplementary Figure S34: Parity plots showing the predictions from the ensemble of artificial neural network (ANN) models trained on the copper MLEARN dataset.

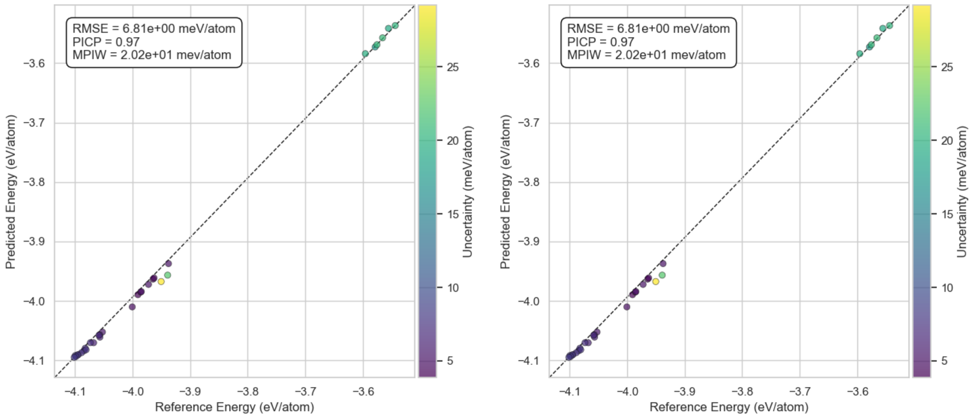

(a) Energy predictions on the test dataset. (b) Force predictions on the test dataset.

Supplementary Figure S35: Parity plots showing the predictions from the Monte Carlo dropout (MCD) model trained on the copper MLEARN dataset.

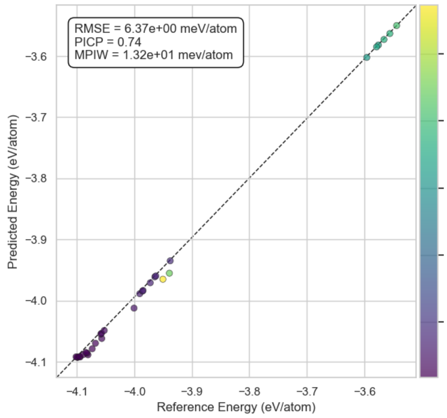

(a) Energy predictions on the test dataset.

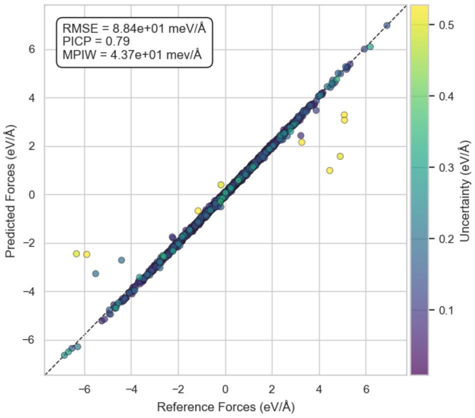

(b) Force predictions on the test dataset.

Supplementary Figure S36: Parity plots showing the predictions from the mean-variance estimation (MVE) model trained on the copper MLEARN dataset.

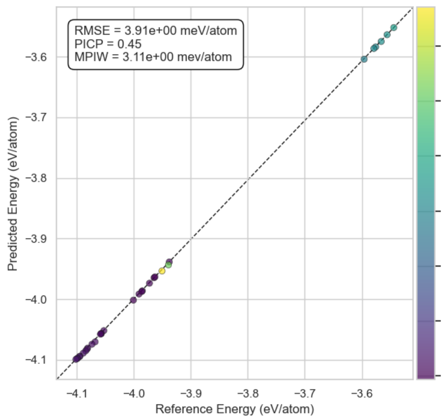

(a) Energy predictions on the test dataset.

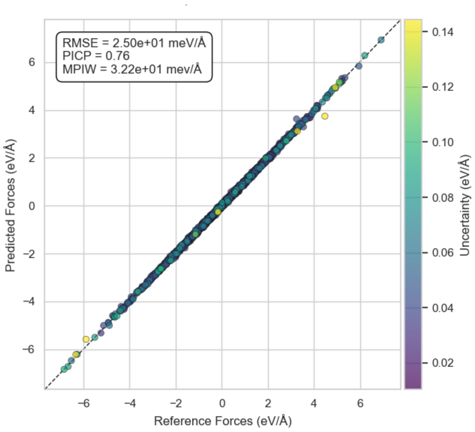

(b) Force predictions on the test dataset.

Supplementary Figure S37: Parity plots showing the predictions from the mixture density network (MDN) model trained on the copper MLEARN dataset.

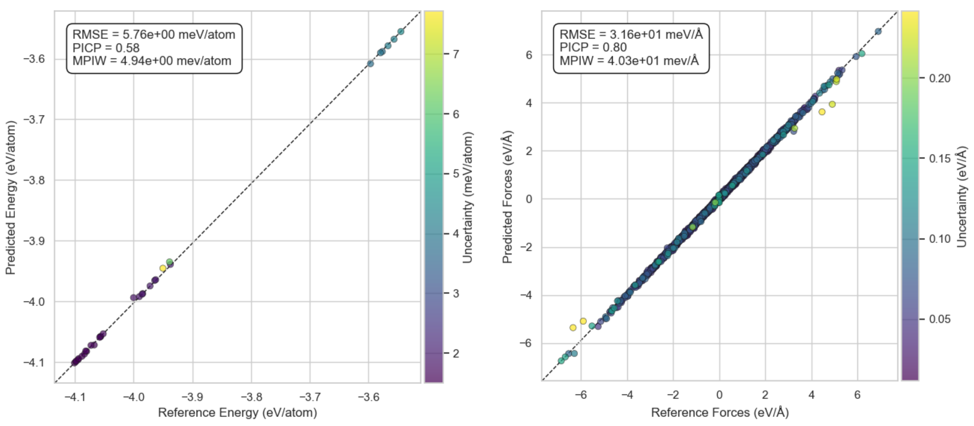

(a) Energy predictions on the test dataset. (b) Force predictions on the test dataset.

Supplementary Figure S38: Parity plots showing the predictions from the Bayesian neural network (BNN) model trained on the copper MLEARN dataset.

6.2 Si MLEARN

Supplementary Figure S39 – Supplementary Figure S43 show the energy and forces parity plots for all model types trained on the silicon MLEARN dataset.

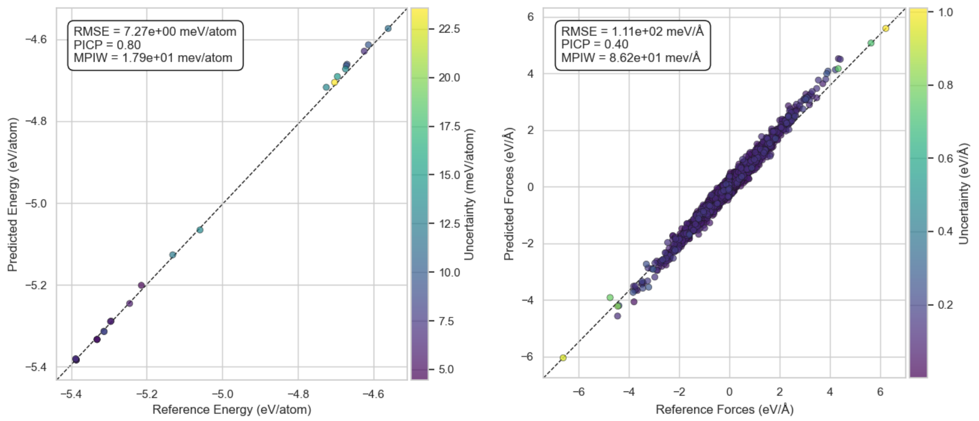

(a) Energy predictions on the test dataset. (b) Force predictions on the test dataset.

Supplementary Figure S39: Parity plots showing the predictions from the ensemble of artificial neural network (ANN) models trained on the silicon MLEARN dataset.

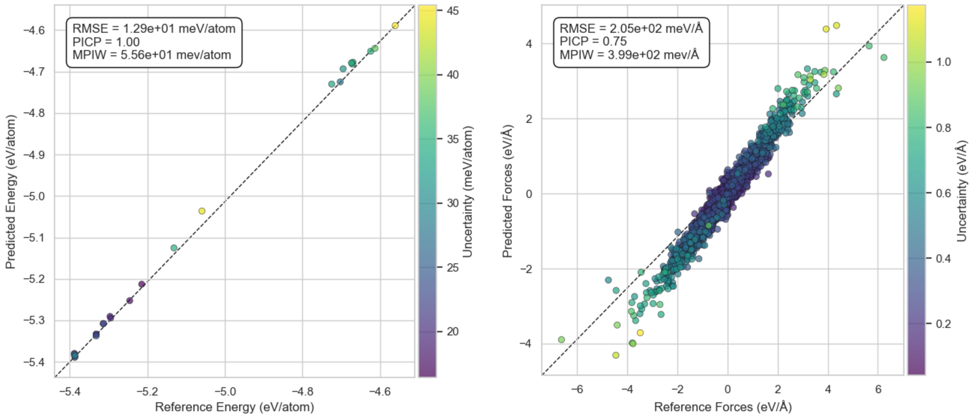

(a) Energy predictions on the test dataset. (b) Force predictions on the test dataset.

Supplementary Figure S40: Parity plots showing the predictions from the Monte Carlo dropout (MCD) model trained on the silicon MLEARN dataset.

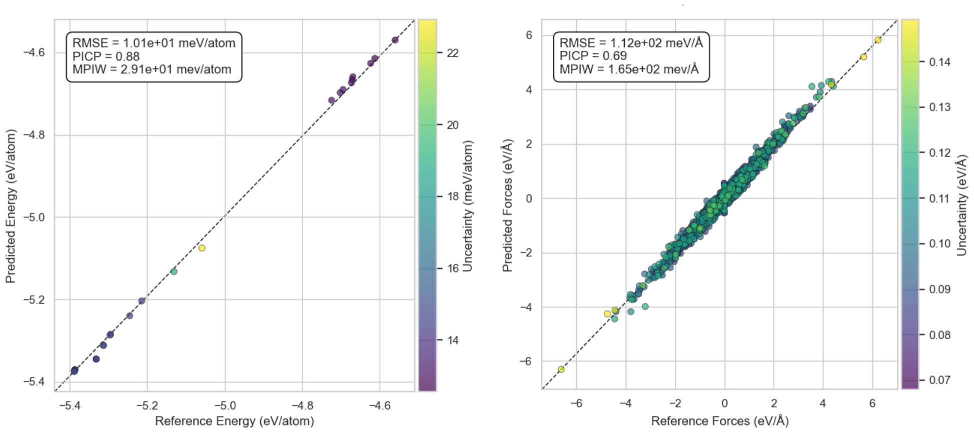

(a) Energy predictions on the test dataset. (b) Force predictions on the test dataset.

Supplementary Figure S41: Parity plots showing the predictions from the mean-variance estimation (MVE) model trained on the silicon MLEARN dataset.

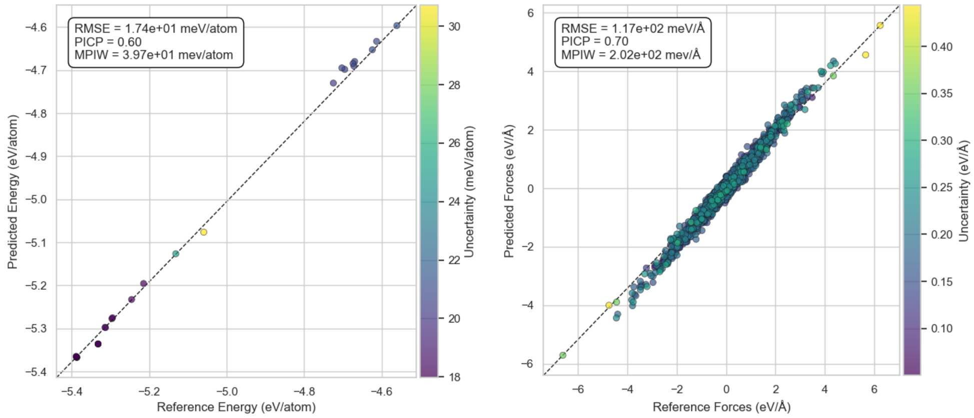

(a) Energy predictions on the test dataset.

(b) Force predictions on the test dataset.

Supplementary Figure S42: Parity plots showing the predictions from the mixture density network (MDN) model trained on the silicon MLEARN dataset.

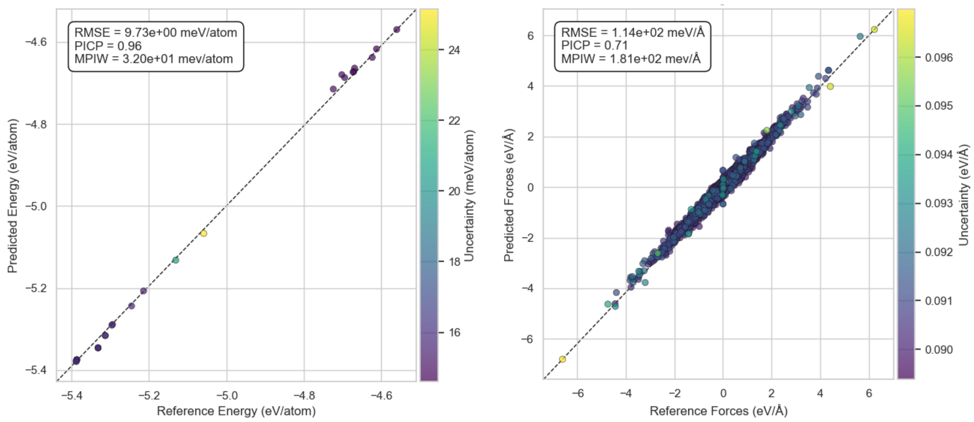

(a) Energy predictions on the test dataset.

(b) Force predictions on the test dataset.

Supplementary Figure S43: Parity plots showing the predictions from the Bayesian neural network (BNN) model trained on the silicon MLEARN dataset.

6.3 Ni MLEARN

Supplementary Figure S44 – Supplementary Figure S48 show the energy and forces parity plots for all model types trained on the nickel MLEARN dataset.

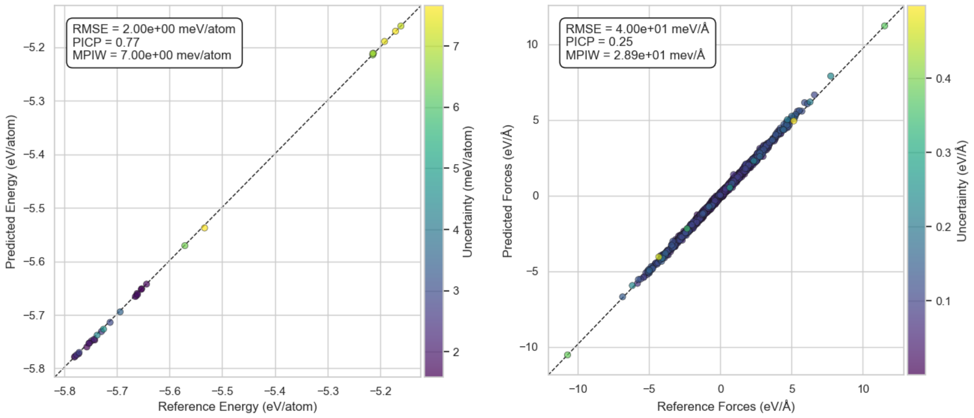

(a) Energy predictions on the test dataset. (b) Force predictions on the test dataset.

Supplementary Figure S44: Parity plots showing the predictions from the ensemble of artificial neural network (ANN) models trained on the nickel MLEARN dataset.

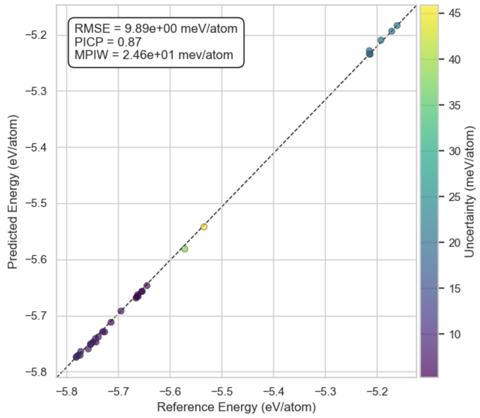

(a) Energy predictions on the test dataset.

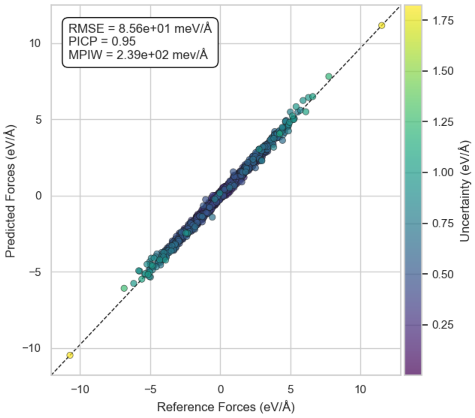

(b) Force predictions on the test dataset.

Supplementary Figure S45: Parity plots showing the predictions from the Monte Carlo dropout (MCD) model trained on the nickel MLEARN dataset.

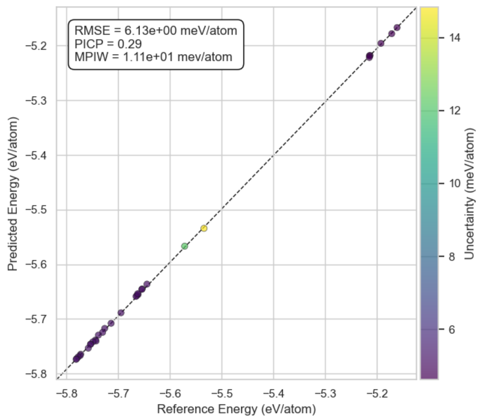

(a) Energy predictions on the test dataset.

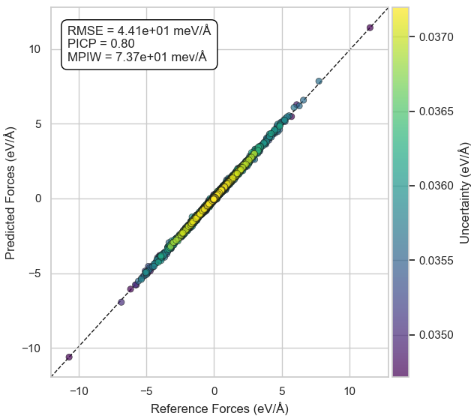

(b) Force predictions on the test dataset.

Supplementary Figure S46: Parity plots showing the predictions from the mean-variance estimation (MVE) model trained on the nickel MLEARN dataset.

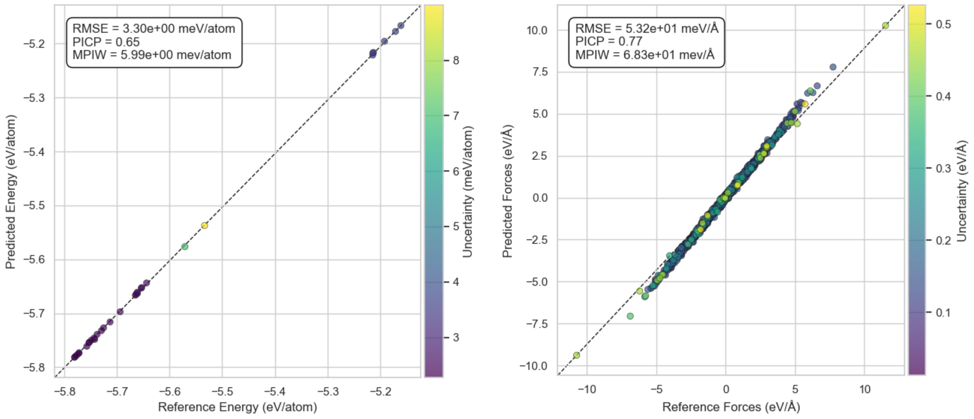

(a) Energy predictions on the test dataset. (b) Force predictions on the test dataset.

Supplementary Figure S47: Parity plots showing the predictions from the mixture density network (MDN) model trained on the nickel MLEARN dataset.

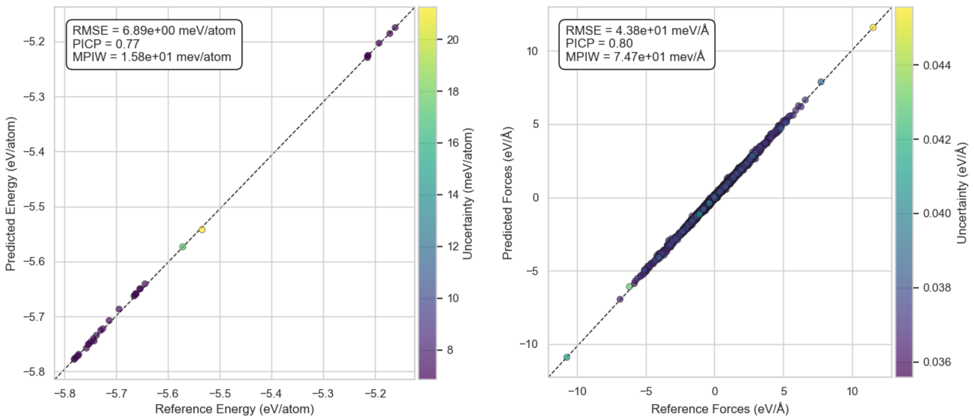

(a) Energy predictions on the test dataset. (b) Force predictions on the test dataset.

Supplementary Figure S48: Parity plots showing the predictions from the Bayesian neural network (BNN) model trained on the nickel MLEARN dataset.

6.4 Mo MLEARN

Supplementary Figure S49 – Supplementary Figure S53 show the energy and forces parity plots for all model types trained on the molybdenum MLEARN dataset.

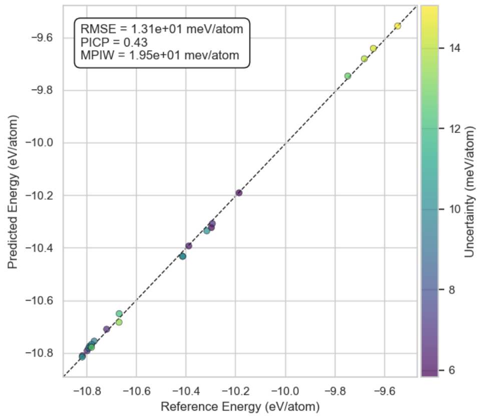

(a) Energy predictions on the test dataset.

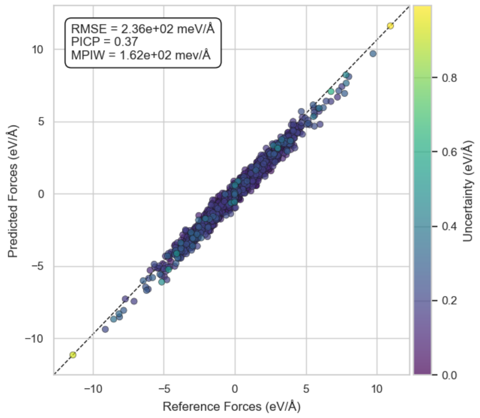

(b) Force predictions on the test dataset.

Supplementary Figure S49: Parity plots showing the predictions from the ensemble of artificial neural network (ANN) models trained on the molybdenum MLEARN dataset.

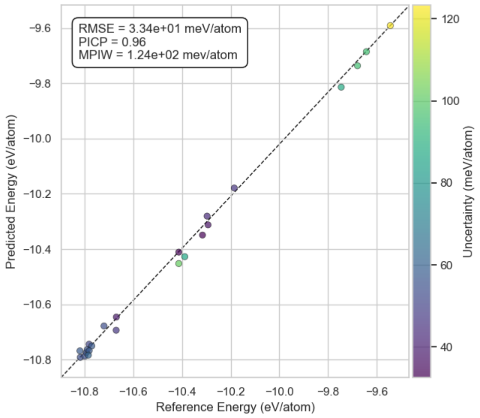

(a) Energy predictions on the test dataset.

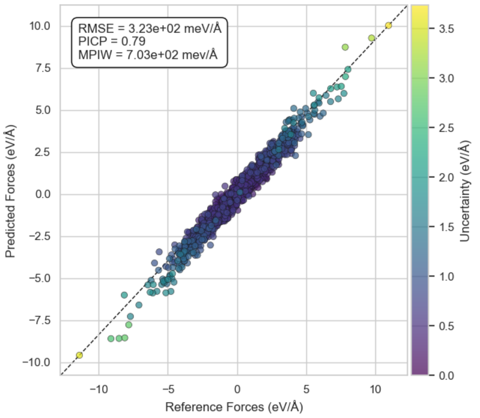

(b) Force predictions on the test dataset.

Supplementary Figure S50: Parity plots showing the predictions from the Monte Carlo dropout (MCD) model trained on the molybdenum MLEARN dataset.

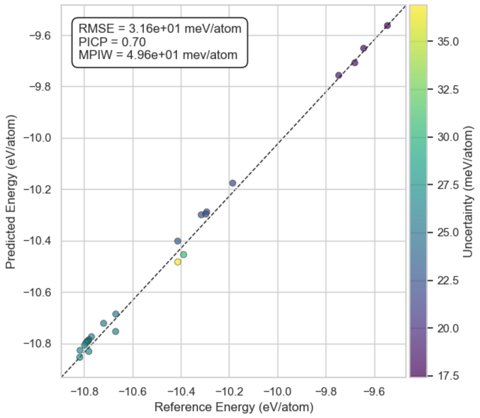

(a) Energy predictions on the test dataset.

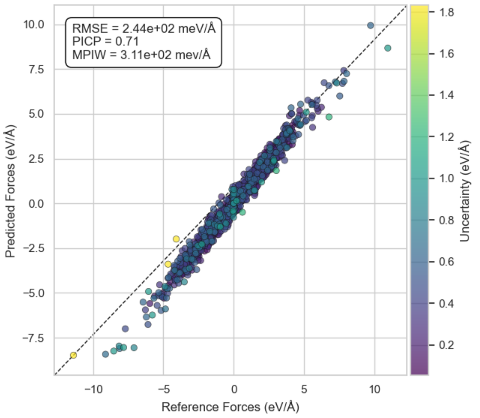

(b) Force predictions on the test dataset.

Supplementary Figure S51: Parity plots showing the predictions from the mean-variance estimation (MVE) model trained on the molybdenum MLEARN dataset.

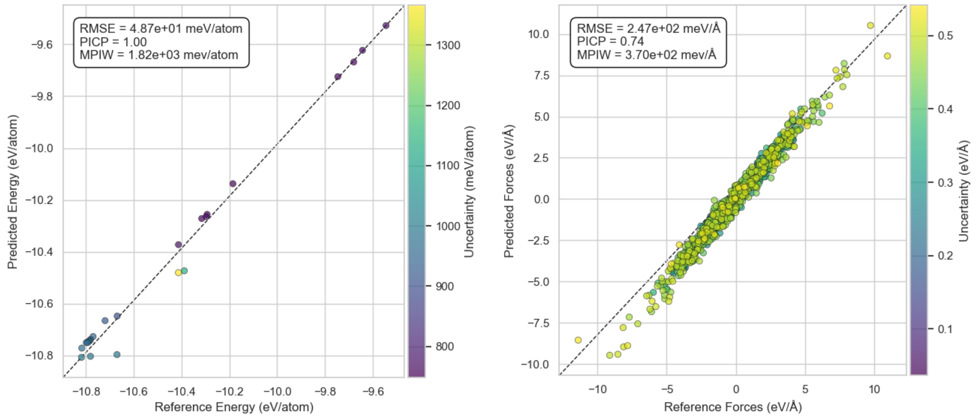

(a) Energy predictions on the test dataset.

(b) Force predictions on the test dataset.

Supplementary Figure S52: Parity plots showing the predictions from the mixture density network (MDN) model trained on the molybdenum MLEARN dataset.

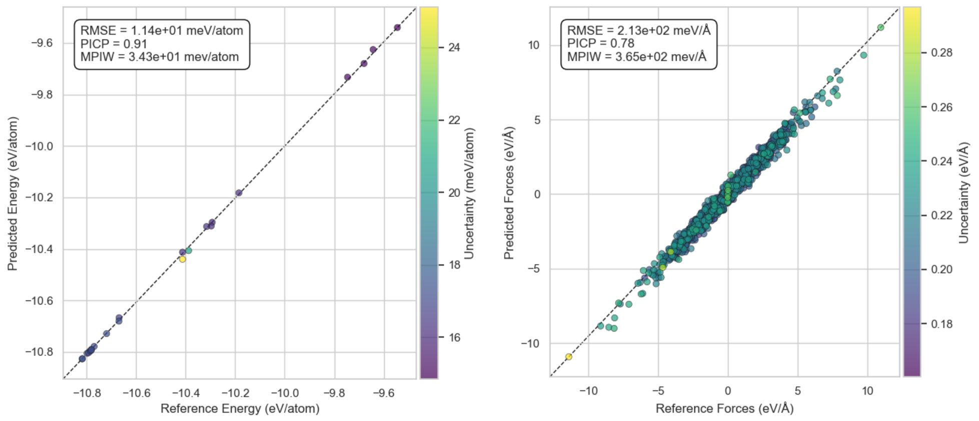

(a) Energy predictions on the test dataset.

(b) Force predictions on the test dataset.

Supplementary Figure S53: Parity plots showing the predictions from the Bayesian neural network (BNN) model trained on the molybdenum MLEARN dataset.

6.5 Li MLEARN

Supplementary Figure S54 – Supplementary Figure S58 show the energy and forces parity plots for all model types trained on the lithium MLEARN dataset.

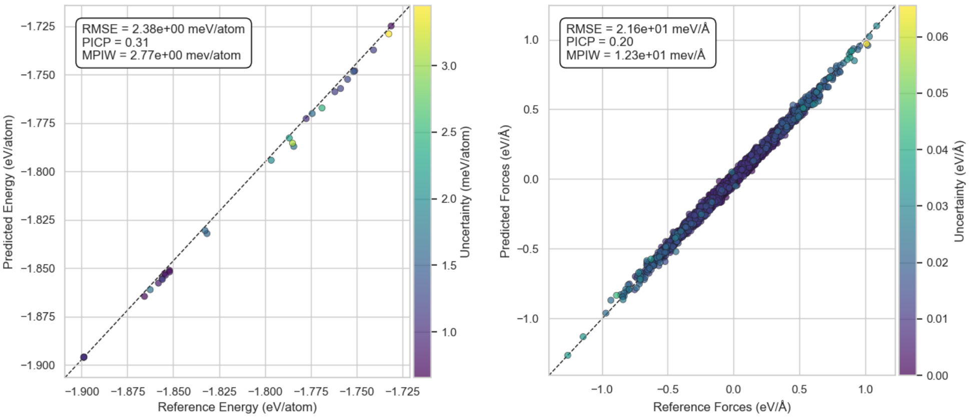

(a) Energy predictions on the test dataset.

(b) Force predictions on the test dataset.

Supplementary Figure S54: Parity plots showing the predictions from the ensemble of artificial neural network (ANN) models trained on the lithium MLEARN dataset.

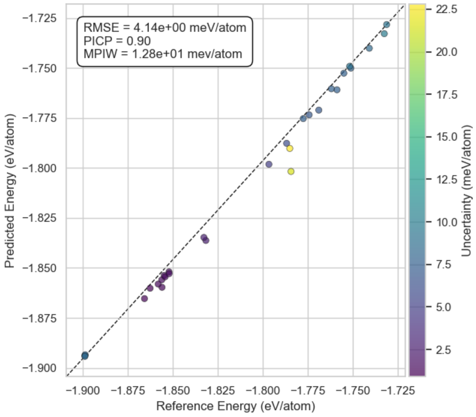

(a) Energy predictions on the test dataset.

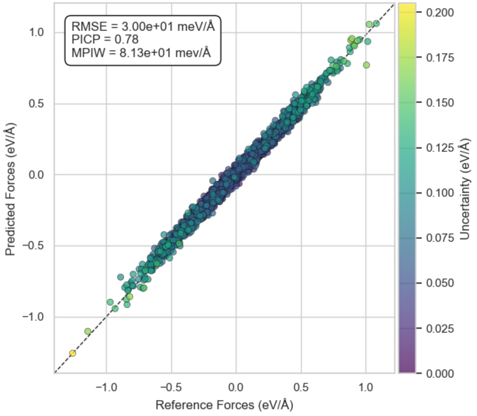

(b) Force predictions on the test dataset.

Supplementary Figure S55: Parity plots showing the predictions from the Monte Carlo dropout (MCD) model trained on the lithium MLEARN dataset.

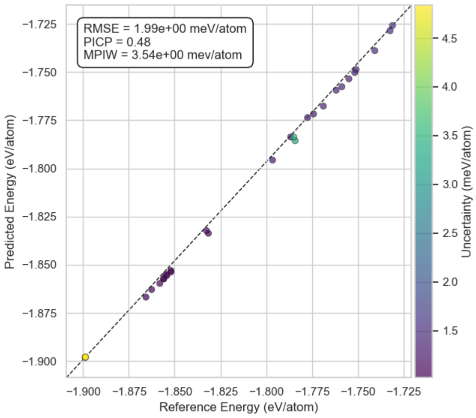

(a) Energy predictions on the test dataset.

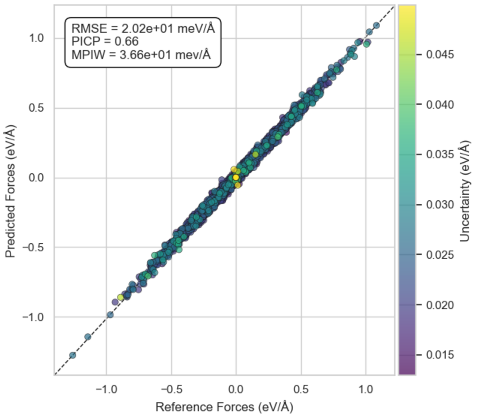

(b) Force predictions on the test dataset.

Supplementary Figure S56: Parity plots showing the predictions from the mean-variance estimation (MVE) model trained on the lithium MLEARN dataset.

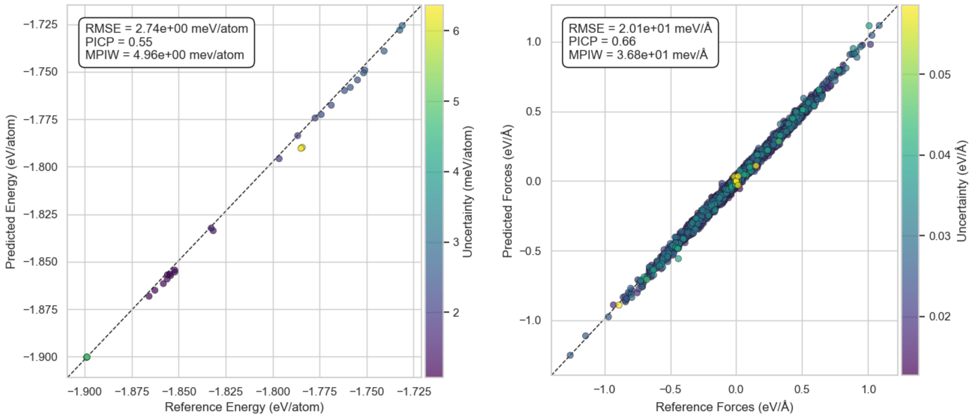

(a) Energy predictions on the test dataset. (b) Force predictions on the test dataset.

Supplementary Figure S57: Parity plots showing the predictions from the mixture density network (MDN) model trained on the lithium MLEARN dataset.

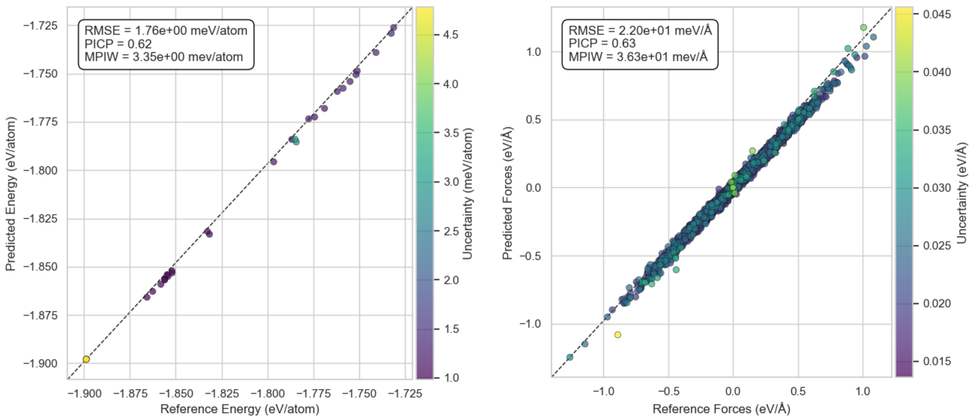

(a) Energy predictions on the test dataset. (b) Force predictions on the test dataset.

Supplementary Figure S58: Parity plots showing the predictions from the Bayesian neural network (BNN) model trained on the lithium MLEARN dataset.

6.6 Ge MLEARN

Supplementary Figure S59 – Supplementary Figure S63 show the energy and forces parity plots for all model types trained on the germanium MLEARN dataset.

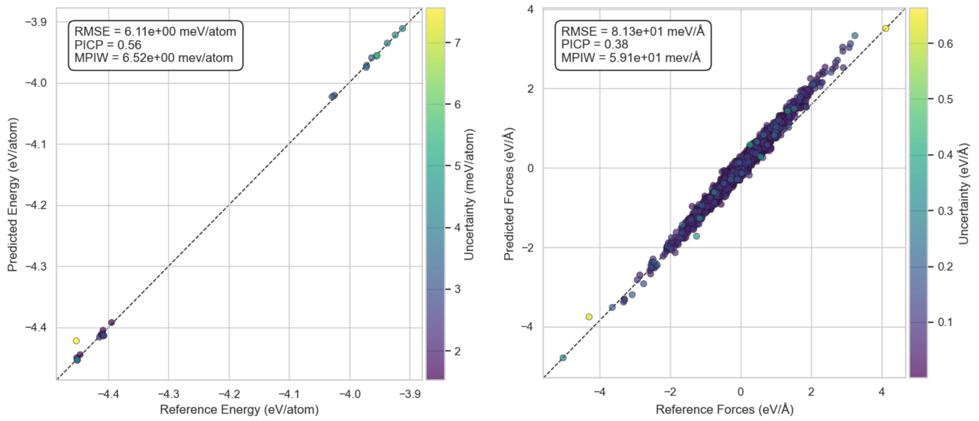

(a) Energy predictions on the test dataset. (b) Force predictions on the test dataset.

Supplementary Figure S59: Parity plots showing the predictions from the ensemble of artificial neural network (ANN) models trained on the germanium MLEARN dataset.

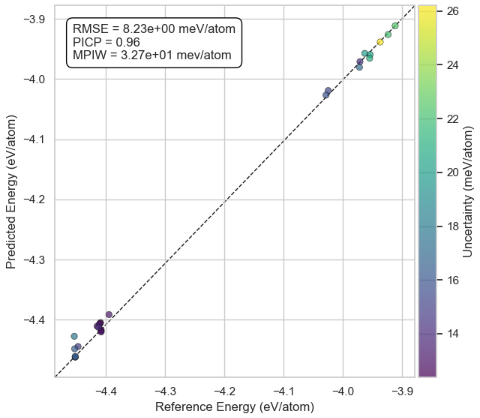

(a) Energy predictions on the test dataset.

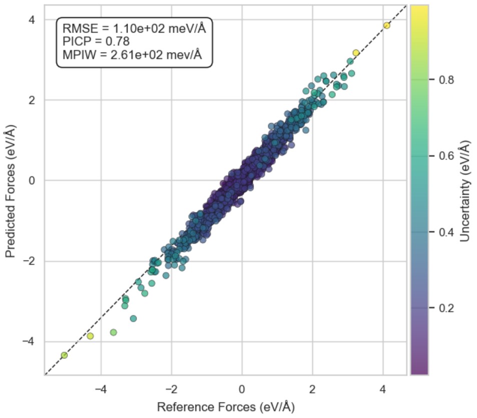

(b) Force predictions on the test dataset.

Supplementary Figure S60: Parity plots showing the predictions from the Monte Carlo dropout (MCD) model trained on the germanium MLEARN dataset.

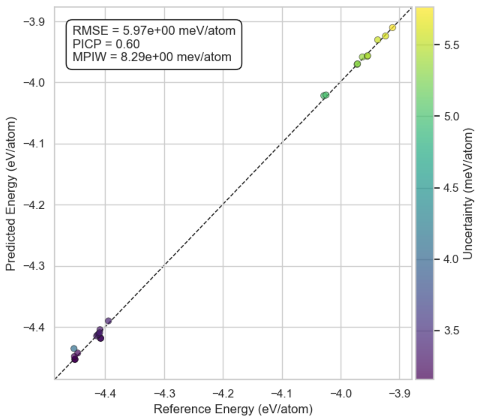

(a) Energy predictions on the test dataset.

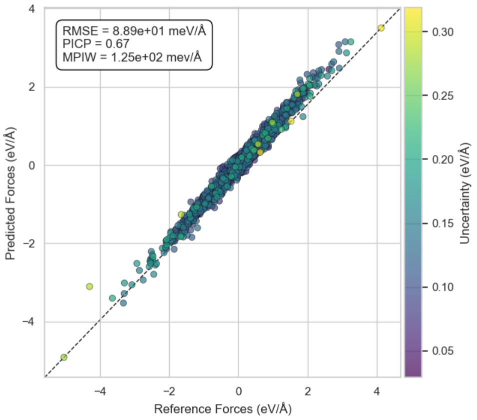

(b) Force predictions on the test dataset.

Supplementary Figure S61: Parity plots showing the predictions from the mean-variance estimation (MVE) model trained on the germanium MLEARN dataset.

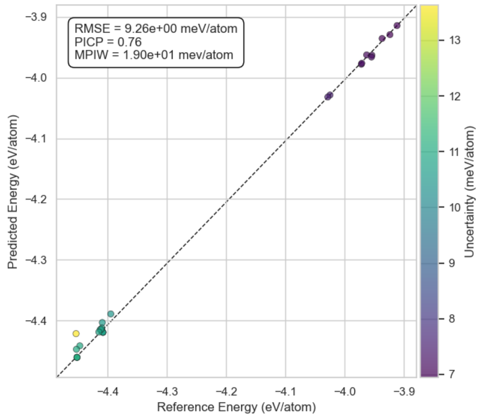

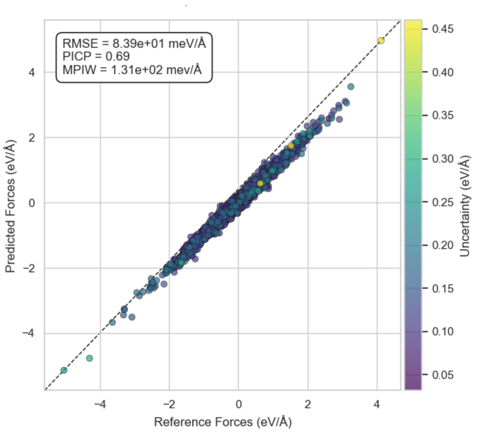

(a) Energy predictions on the test dataset.

(b) Force predictions on the test dataset.

Supplementary Figure S62: Parity plots showing the predictions from the mixture density network (MDN) model trained on the germanium MLEARN dataset.

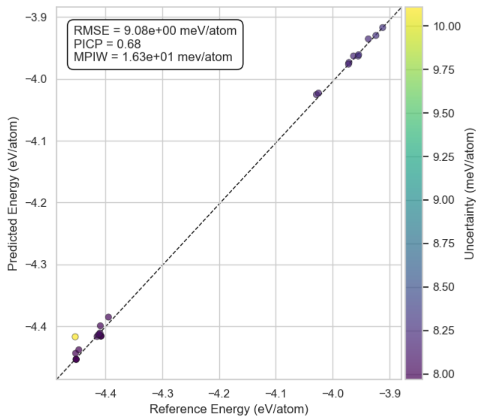

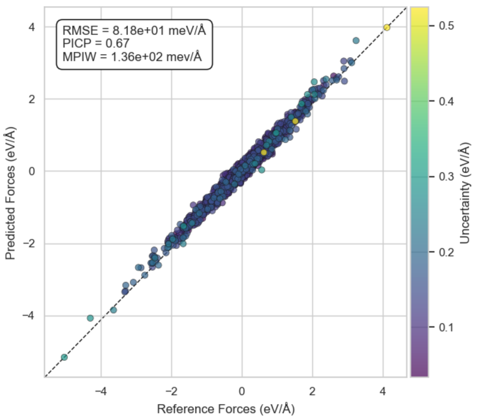

(a) Energy predictions on the test dataset.

(b) Force predictions on the test dataset.

Supplementary Figure S63: Parity plots showing the predictions from the Bayesian neural network (BNN) model trained on the germanium MLEARN dataset.

6.7 GAP-20 Dataset

Supplementary Figure S64 – Supplementary Figure S68 show the energy and forces parity plots for all model types trained on the carbon GAP-20 dataset.

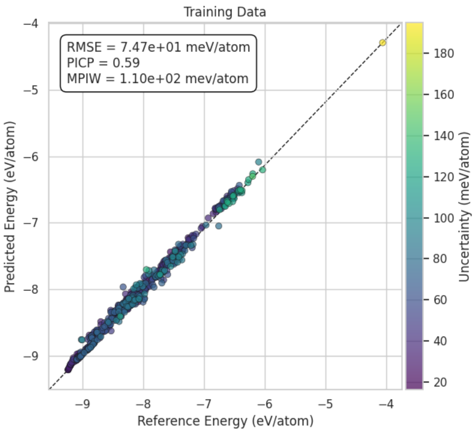

(a) Energy predictions on the test dataset.

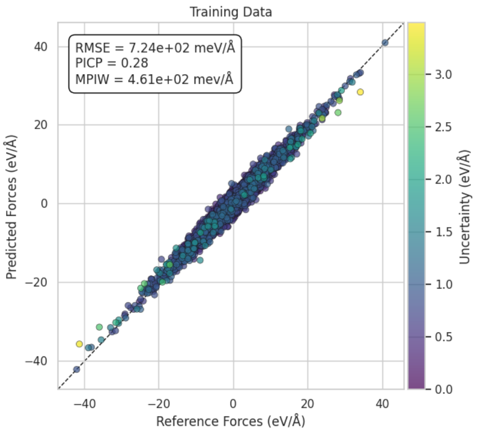

(b) Force predictions on the test dataset.

Supplementary Figure S64: Parity plots showing the predictions from the ensemble of artificial neural network (ANN) models trained on the carbon GAP-20 dataset.

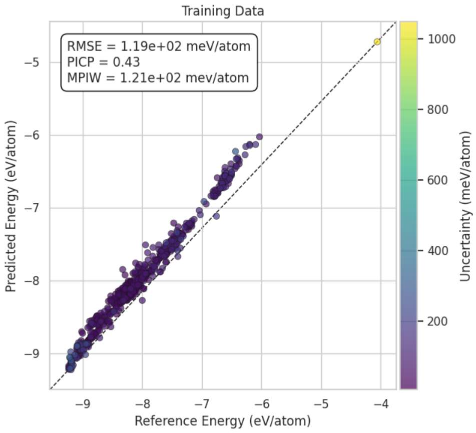

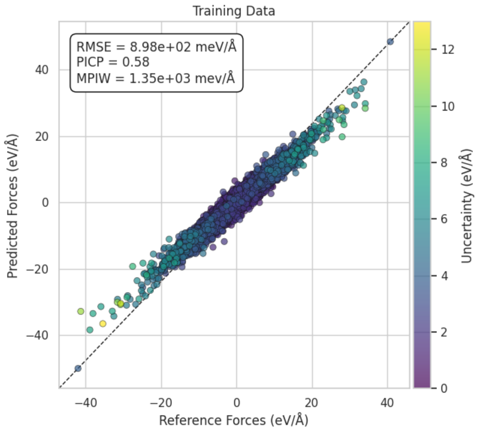

(a) Energy predictions on the test dataset. (b) Force predictions on the test dataset.

Supplementary Figure S65: Parity plots showing the predictions from the Monte Carlo dropout (MCD) model trained on the carbon GAP-20 dataset.

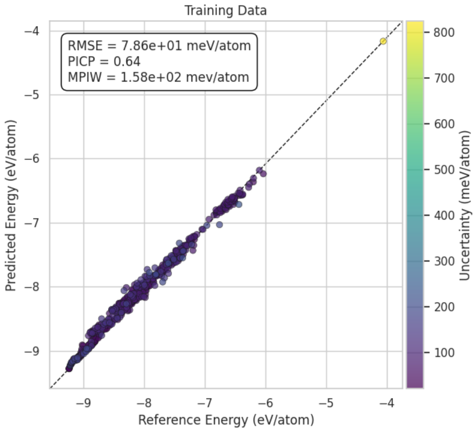

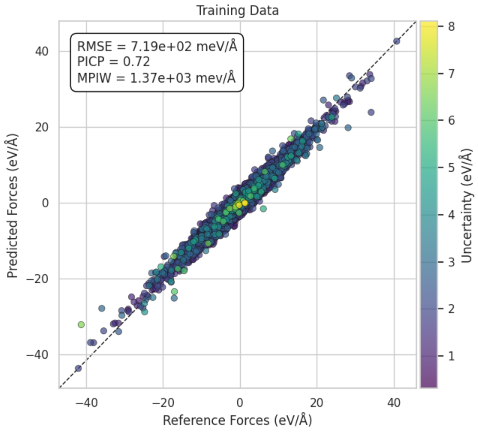

(a) Energy predictions on the test dataset. (b) Force predictions on the test dataset.

Supplementary Figure S66: Parity plots showing the predictions from the mean-variance estimation (MVE) model trained on the carbon GAP-20 dataset.

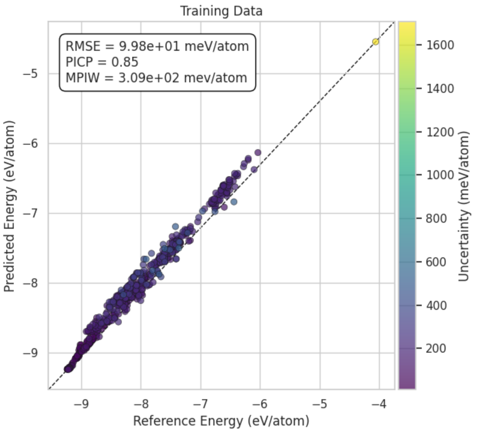

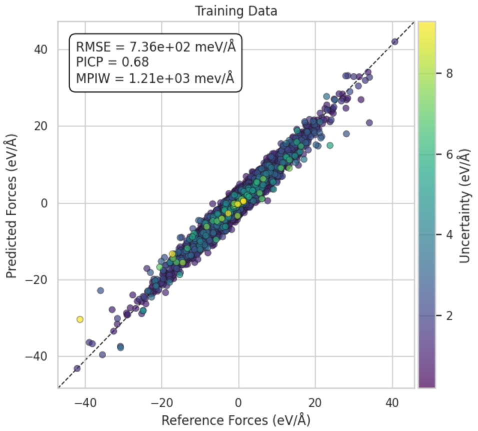

(a) Force predictions on the test dataset. (b) Force predictions on the test dataset.

Supplementary Figure S67: Parity plots showing the predictions from the mixture density network (MDN) model trained on the carbon GAP-20 dataset.

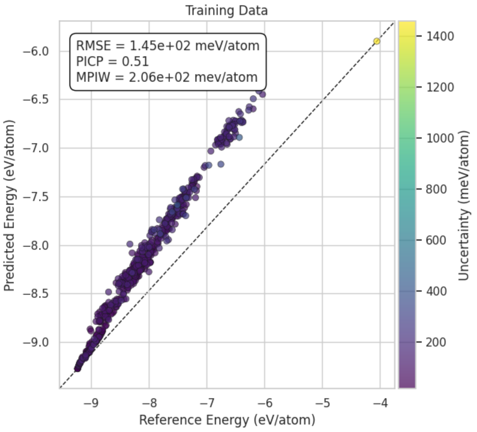

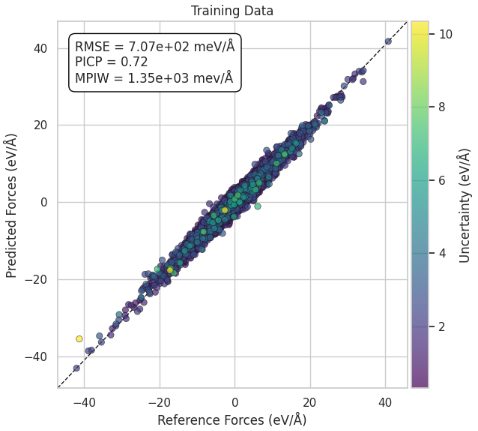

(a) Energy predictions on the test dataset. (b) Force predictions on the test dataset.

Supplementary Figure S68: Parity plots showing the predictions from the Bayesian neural network (BNN) model trained on the carbon GAP-20 dataset.
